# Extraction Optimization and Preliminary Neuroprotective Evaluation of Asiaticoside and Asiatic Acid from *Centella asiatica* (L.) Urb Using In Vitro and In Silico Approaches

**DOI:** 10.3390/ijms27208951

**Published:** 2026-10-09

**Authors:** Chaiyasit Winyuchakhrit, Sarin Tadtong, Chatchapong Tangjidapichai, Weerasak Samee

**Affiliations:** Faculty of Pharmacy, Srinakharinwirot University, Nakhon Nayok 26120, Thailand; chaiyasit.winyu@g.swu.ac.th (C.W.); sarin@g.swu.ac.th (S.T.); chatchapong@g.swu.ac.th (C.T.)

**Keywords:** asiatic acid, extraction, sustainable, HPLC, neuroprotective, antioxidant, anti-inflammatory, docking

## Abstract

Neurodegenerative diseases are closely associated with chronic neuroinflammation and oxidative stress. *Centella asiatica* (L.) Urb. is a medicinal plant with recognized cognitive-enhancing and neuroprotective properties. This study optimized the extraction of asiaticoside (AS) and asiatic acid (AA) using an aqueous–ethanol system guided by Hansen solubility parameters (HSP) and evaluated their antioxidant and neuroprotective activities through in vitro and in silico approaches. HSP predictions identified 60% ethanol as the optimal solvent, yielding 9.94 ± 0.24 mg/g dry weight of AS and 3.26 ± 0.07 mg/g dry weight of AA. The extraction method demonstrated favorable sustainability and applicability, with AGREEprep™ and Blue Applicability Grade Index scores of 0.68 and 72.5, respectively. The 60% ethanol extract contained 7.61 ± 0.24 mg gallic acid equivalents/g total phenolics and 22.64 ± 3.71 mg quercetin equivalents/g total flavonoids and exhibited 2,2-diphenyl-1-picrylhydrazyl (DPPH) radical-scavenging activity with an IC_50_ of 0.12 ± 0.01 mg/mL. In an Aβ_42_-induced (Amyloid-β42) SH-SY5Y cell model, the extract, AS, and AA increased cell viability to 80.21 ± 10.62%, 75.92 ± 2.43%, and 92.81 ± 1.90%, respectively. ELISA and molecular docking analyses indicated putative interactions with TNF-α, IL-18, HO-1 and caspase-3. AS and AA are predicted to be involved in modulating neuroinflammation, oxidative stress, and apoptosis, suggesting their potential neuroprotective applications.

## 1. Introduction

*Centella asiatica* (L.) Urb., commonly known as gotu kola, is a herbaceous plant belonging to the family Apiaceae. Native to humid tropical and subtropical regions, it has been widely used as a medicinal herb in Ayurvedic and traditional Chinese medicine for centuries [1]. The leaves of *C. asiatica* have been extensively studied because of their diverse pharmacological properties, including wound-healing, dermatological, antioxidant, anti-inflammatory, memory-enhancing, and neuroprotective effects [2]. The anti-inflammatory activity of *C. asiatica* has been demonstrated by its ability to reduce the production of interleukin-1β (IL-1β), interleukin-6 (IL-6), tumor necrosis factor-α (TNF-α), and prostaglandin E2 (PGE2), as well as to inhibit cyclooxygenase-2 (COX-2) and lipoxygenase (LOX) activity [3,4,5]. In addition, computational approaches, particularly molecular docking, can be used to characterize the interactions between *C. asiatica* constituents and specific molecular targets. While molecular docking serves as a valuable predictive tool to explore potential mechanisms of action, it remains a computational model that requires experimental validation to confirm biological relevance [6,7,8].

**Figure 1:**
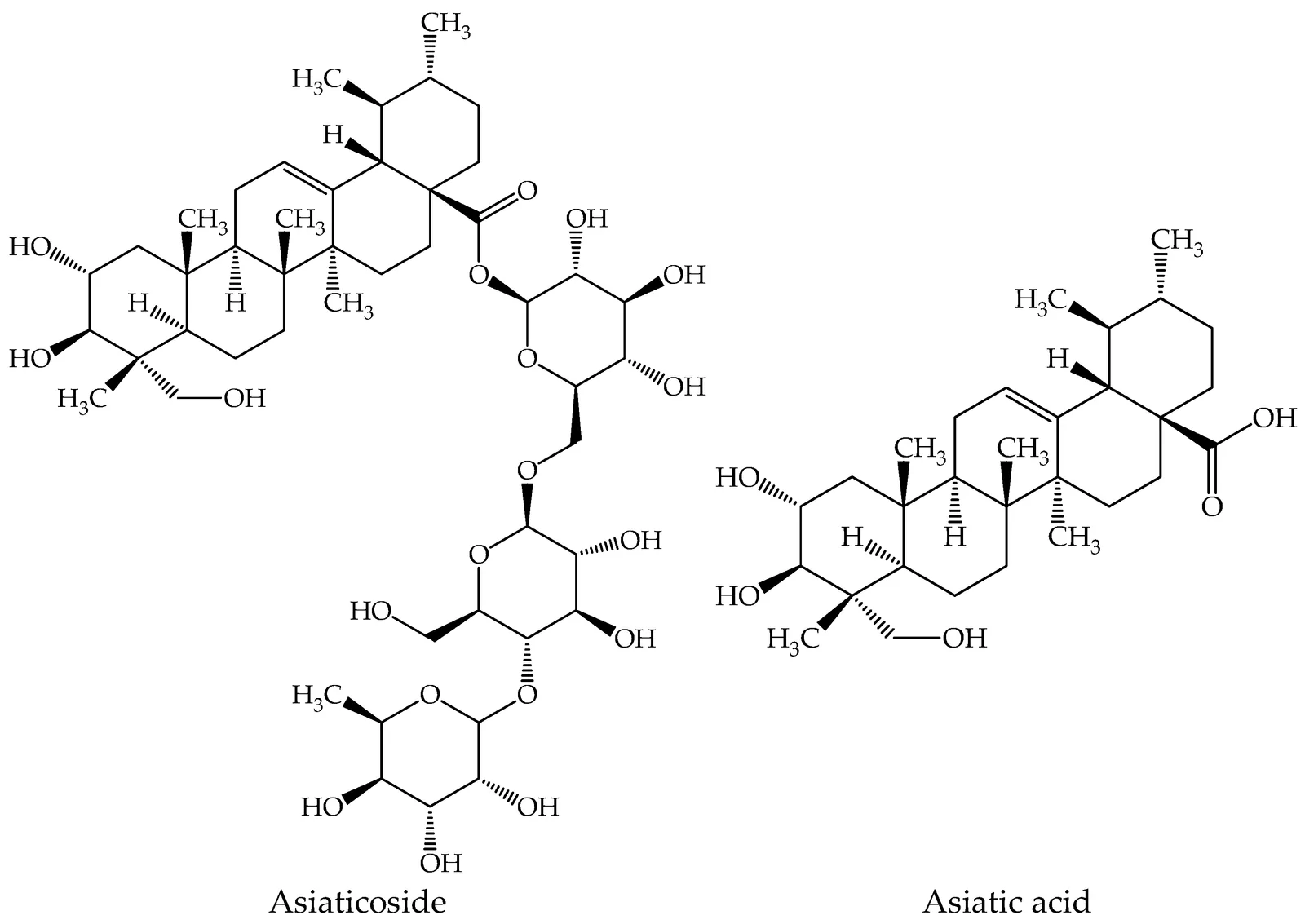
Chemical structures of asiaticoside and asiatic acid.

The pharmaceutical value of *C. asiatica* and its potential application in health-promoting products are largely attributed to its abundant bioactive triterpenoids [2,9]. Among these constituents, asiatic acid (AA) and asiaticoside (AS) have been strongly associated with the plant’s neuroprotective effects [10,11,12]. Consequently, there is increasing demand for high-quality *C. asiatica* extracts containing standardized and reproducible concentrations of these bioactive compounds, particularly for industrial-scale applications. However, the simultaneous extraction of AS and AA remains challenging because of their substantially different physicochemical properties. AS is a glycoside containing a sugar moiety and is therefore more polar than AA, whereas AA is an aglycone with lower polarity. AA is predominantly lipophilic and exhibits limited water solubility (Figure 1). This marked difference in polarity can limit the efficiency of conventional extraction procedures for the concurrent recovery of both compounds [13,14].

Conventional methods for extracting bioactive constituents from *C. asiatica* commonly involve maceration with aqueous ethanol, such as 75% ethanol, or Soxhlet extraction. Nevertheless, these techniques often provide limited selectivity toward the target compounds, require prolonged processing times, consume considerable quantities of solvents, and may result in insufficient extraction yields [15,16]. Furthermore, the use of certain organic solvents may raise toxicological and environmental concerns [17,18]. Advanced extraction techniques, including ultrasound-assisted extraction (UAE), have been applied to improve extraction efficiency [19,20]. However, the enhanced performance of these methods is frequently achieved through increased energy input rather than through the rational consideration of the compound-specific physicochemical properties that govern extraction selectivity [21,22].

Hansen solubility parameters (HSPs) offer a systematic framework for the rational selection of solvents and optimization of extraction processes. HSP theory describes intermolecular interactions using three components: the dispersion parameter (δD), the polar parameter (δP), and the hydrogen-bonding parameter (δH). Based on the principle of “like dissolves like,” HSPs enable the prediction and selection of individual solvents or solvent mixtures with solubility characteristics that closely match those of the target compounds, thereby improving extraction efficiency and yield [23]. HSP-guided solvent selection can reduce the need for extensive empirical screening, minimize solvent consumption, shorten processing times, and decrease research and development costs. Moreover, HSP theory has increasingly been applied to the design of environmentally preferable solvent systems, supporting the development of selective extraction procedures while reducing dependence on potentially hazardous organic solvents [24,25].

In addition to extraction performance, the environmental sustainability and practical applicability of analytical procedures should also be considered. AGREEprep™ (Analytical Greenness Metric for Sample Preparation, Gdańsk, Poland) is a quantitative tool used to evaluate the environmental sustainability and safety of sample preparation procedures. The assessment is based on the ten principles of green sample preparation. Individual criteria are scored on a scale from 0 to 1, where 0 represents the least favorable or most hazardous condition and 1 represents the most favorable and environmentally benign condition. The weighted criteria are subsequently combined to generate an overall greenness score, which is presented as a color-coded pictogram resembling a traffic-light system [26,27]. Blue Applicability Grade Index (BAGI) is a complementary metric that evaluates the practical applicability, efficiency, speed, and cost-effectiveness of analytical methods. It represents the “blue” dimension of White Analytical Chemistry, which emphasizes operational simplicity and suitability for routine laboratory use. BAGI assesses ten key aspects of an analytical procedure and provides a score ranging from 25 to 100. Higher scores indicate greater practicality, efficiency, user-friendliness, and suitability for routine analytical applications [28,29].

Frontier molecular orbital (FMO) theory provides a useful framework for understanding the electronic factors that influence antioxidant activity, particularly the energies of the highest occupied molecular orbital (HOMO) and the lowest unoccupied molecular orbital (LUMO). The HOMO represents the ability to donate electrons, whereas the LUMO reflects its capacity to accept them. A smaller HOMO–LUMO energy gap generally corresponds to greater chemical reactivity and may therefore enhance antioxidant performance by facilitating electron transfer [30,31]. Molecular features such as hydroxyl groups, conjugated double bonds, and extensive electron delocalization also contribute to molecular stability and strongly influence the compound’s antioxidant potential [24].

In this study, HSP theory was integrated with experimental aqueous–ethanol extraction to predict and select an environmentally preferable solvent system for the simultaneous extraction of AS and AA from *C. asiatica*. The optimized extract was characterized in terms of its total phenolic content (TPC) and total flavonoid content (TFC), and its antioxidant, anti-inflammatory, and neuroprotective activities were evaluated. The neuroprotective effects of the extract, AS, and AA were further investigated using the in vitro assays. Molecular docking was employed to explore the binding interactions of AS and AA with selected targets associated with inflammation (TNF-α and IL-18), apoptosis (caspase-3) and oxidative stress (heme oxygenase (HO-1)). Finally, the environmental sustainability and practical applicability of the sample preparation procedure were assessed using the AGREEprep™ and BAGI metrics. This integrated strategy aims to provide a rational and comprehensive approach for developing an efficient, sustainable, and biologically relevant extraction platform for neuroprotective compounds from *C. asiatica*.

## 2. Results

### 2.1. Physicochemical Properties of Asiaticoside and Asiatic Acid

**Figure 2:**
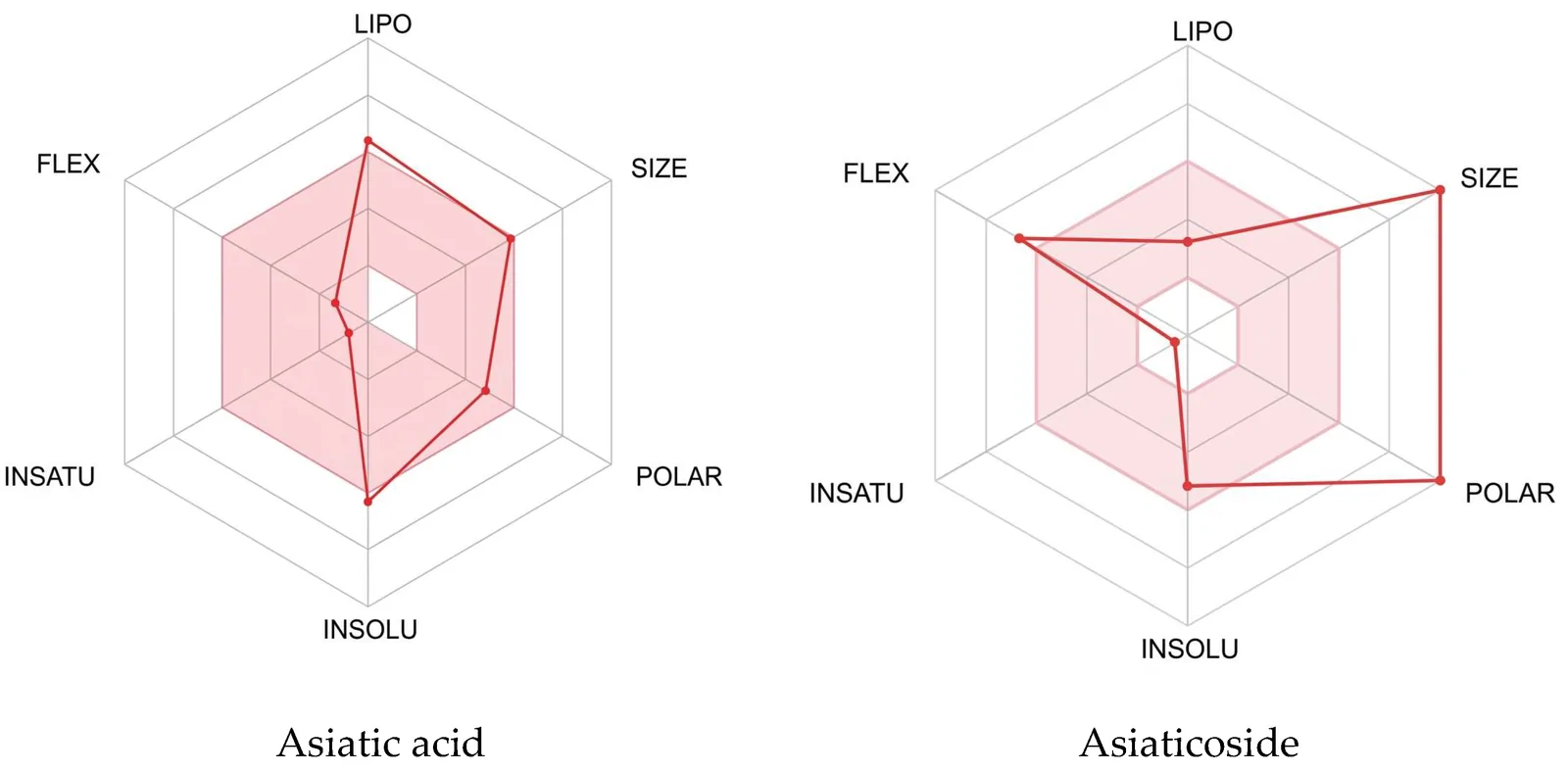
Pink zone oral bioavailability radar for asiaticoside and asiatic acid. The red line represents the physicochemical properties of the compounds.

AS is a trisaccharide glycoside derived from AA. These structural differences confer distinct physicochemical properties. The calculated logP and topological polar surface area (TPSA) values obtained using SwissADME suggest that AS is more hydrophilic and potentially more water-soluble than AA [32]. However, as shown in Table 1, AA is predicted to have low gastrointestinal (GI) absorption, whereas AS is predicted to have negligible GI absorption. This poor absorption is likely attributable to its violation of three physicochemical criteria of Lipinski’s rule: a molecular weight greater than 500 g/mol, more than 10 hydrogen-bond acceptors, and more than 5 hydrogen-bond donors. As shown in Figure 2, the pink region of the diagram indicates that AS has a relatively large molecular size and high polar surface area, both of which may limit its passive gastrointestinal permeability.

### 2.2. Identification of Asiaticoside and Asiatic Acid in Centella asiatica Leaves Extract

**Figure 3:**
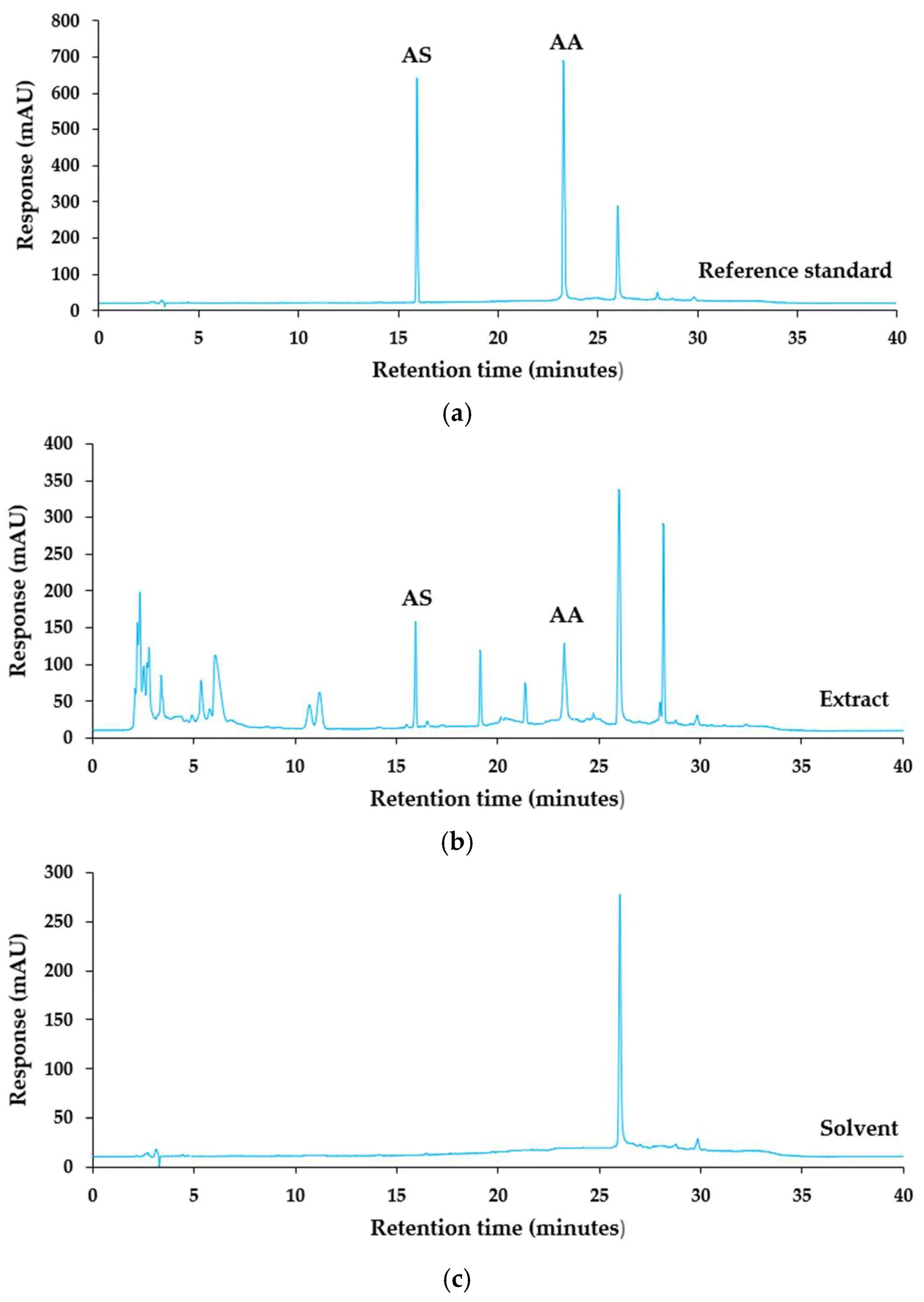
Chromatograms of (**a**) reference standards—asiaticoside (AS) and asiatic acid (AA); (**b**) *C. asiatica* leaf extract; and (**c**) solvent, recorded at 210 nm.

High-performance liquid chromatography coupled with photodiode array detection (HPLC–PDA) was employed for the identification of AS and AA in *C. asiatica* samples. The principal peaks detected in the leaf extract were assigned by comparing their retention times and maximum absorbance at 210 nm with those of authentic reference standards. As illustrated in Figure 3, AS and AA exhibited retention times of approximately 15.9 and 23.3 min, respectively. This elution order is consistent with their relative polarities, as reported in Table 1 and previous studies [33].

### 2.3. Validation of HPLC Analytical Method for Determination of Asiaticoside and Asiatic Acid in Centella asiatica Leaf Extracts

The analytical method for the simultaneous determination of AS and AA in *C. asiatica* leaf extracts was developed according to the procedure described in Section 4.4. Its suitability and reliability were assessed through system suitability testing and method validation in accordance with the guidelines of the International Council for Harmonisation (ICH) [34] and the AOAC (2016) [35].

A 60% ethanol extract of *C. asiatica* leaves was analyzed under the specified high-performance liquid chromatography (HPLC) conditions using an injection volume of 10 µL. Six replicate injections were performed. The system suitability results for all analytes complied with the predefined acceptance criteria, with relative standard deviations (%RSDs) for retention time and peak area below 2%. The number of theoretical plates exceeded 2500 in all injections, whereas the tailing factor remained below 1.19 (Table 2). Theoretical plate number is an important indicator of column efficiency, with higher values generally reflecting superior separation performance and sharper, better-resolved analyte peaks. Furthermore, the chromatographic resolution was greater than 2.0, confirming adequate separation between the analytes.

The HPLC–PDA method was validated according to AOAC guidelines by evaluating its linearity, limits of detection (LOD) and quantification (LOQ), precision, and accuracy. Linearity was assessed using five-point calibration curves, with the concentration ranges selected based on the anticipated levels of the analytes in the *C. asiatica* extracts, as established through preliminary studies.

Because the concentrations of AS and AA differ in the herbal extract, different linearity ranges were used for each compound. As shown in Table 3, all reference standards exhibited excellent linearity across the concentration ranges of 32–1290 µg/mL for AS and 32–180 µg/mL for AA, with coefficients of determination (R^2^) between 0.9995 and 0.9998. The limits of detection (LOD) were 10.00 µg/mL for AS and 5.41 µg/mL for AA, while the corresponding limits of quantification (LOQ) were 30.29 and 16.40 µg/mL, respectively.

As shown in Table 4, the recovery of AS ranged from 97.15% to 98.15%, whereas that of AA ranged from 99.55% to 101.67%. These results fell within the acceptable recovery range of 97–103% established by the AOAC Guideline (2016) [35], demonstrating the accuracy of the method. Precision was evaluated using the relative standard deviation (RSD), and the RSD values for repeatability, intra-day precision, and inter-day precision were below 2.00% for both analytes. Thus, the method met the predefined precision criteria. Overall, the validation results confirmed that the developed HPLC assay was accurate, precise, and appropriate for the quantitative determination of AS and AA in *C. asiatica* leaf extracts for quality-control applications.

### 2.4. Solvent Selection for the Extraction of Asiaticoside and Asiatic Acid in Centella asiatica

The efficient extraction of AS and AA from *C. asiatica* depends on the careful selection of an appropriate solvent system to maximize extraction yield while minimizing toxicity. In addition to extraction efficiency, environmental impact and solvent sustainability are important considerations. Solvent selection is influenced by several factors, including the polarity of the target compounds, the efficiency of the extraction technique, and the physicochemical properties of the solvents employed. In the present study, different ethanol–water ratios were investigated in conjunction with ultrasound-assisted extraction.

The Hansen solubility parameter (HSP) model describes solvent–solute interactions in terms of three components: dispersion forces (δd), polar interactions (δp), and hydrogen bonding (δh). These parameters provide a theoretical basis for evaluating the ability of various solvents to dissolve and extract AS and AA. As presented in Table 5, the calculated δd, δp, and δh values for AS were 31.4715, 0.5180, and 37.4938, respectively. For AA, the corresponding values were 20.0773, −3.2630, and 5.9848, respectively, as shown in Table 6.

The Hansen solubility parameter distance (Ra) between a solute and solvent is an important consideration in the selection of an appropriate extraction solvent. This parameter is calculated from the differences in dispersion, polar, and hydrogen-bonding interactions between the solute and solvent. In general, a lower Ra value indicates greater mutual solubility and stronger intermolecular interactions, although extraction efficiency is also affected by solvent polarity, matrix properties, and process conditions.

As shown in Table 7, the Ra values between AS and water and between AS and ethanol were 35.82 and 37.17, respectively. The ethanol–water mixtures exhibited lower Ra values than the pure solvents, with values of 34.67 for 60% ethanol and 34.39 for 40% ethanol. These results suggest that AS may have favorable solubility in intermediate ethanol–water mixtures, particularly those containing 40–60% ethanol. For AA, the Ra values for water and ethanol were 42.11 and 19.97, respectively, indicating a substantially greater affinity for ethanol. The corresponding Ra values for 60% and 80% ethanol were 28.47 and 24.11, respectively, suggesting that ethanol-rich solvents may be favorable for AA extraction.

To assess the relationship between predicted solvent affinity and actual extraction efficiency, a comparative extraction experiment was performed using ethanol–water mixtures containing 100%, 80%, 70%, 60%, 40%, and 20% ethanol, together with water alone (0% ethanol). Dried *C. asiatica* leaf powder was extracted in triplicate (*n* = 3) under each solvent condition at a solid-to-liquid ratio of 1:10 (*w*/*v*). The samples were sonicated for 30 min at 40 kHz and 300 W. The resulting AS and AA contents were then quantified and compared with the corresponding Ra values and dielectric constants.

For AS, 60% ethanol produced the highest extraction yield, at 9.94 ± 0.24 mg/g of dried powder. This was followed by 70%, 40%, 80%, and 100% ethanol, which yielded 9.08 ± 0.38, 8.72 ± 0.11, 8.59 ± 0.99, and 4.83 ± 0.30 mg/g, respectively. Although 40% ethanol exhibited the lowest Ra value for AS (34.39), 60% ethanol provided the highest extraction yield. The latter solvent system had an Ra value of 34.67 and a dielectric constant of 49.81. This finding indicates that the highest extraction efficiency was not determined solely by the minimum Ra value. Instead, it likely resulted from a favorable balance between solvent–solute interactions, solvent polarity, and the effective penetration of the solvent into the plant matrix. AS was not detected in extracts obtained with 20% ethanol or water.

The dielectric constant of the ethanol–water mixtures increased progressively with increasing water content, ranging from 24.30 for 100% ethanol to 80.00 for water. Despite the high dielectric constant of highly aqueous solvents, these systems were ineffective for AS extraction. This result further demonstrates that dielectric constant alone cannot fully predict extraction performance and should be interpreted together with Ra values and the physicochemical characteristics of the target compound.

A similar extraction pattern was observed for AA. The highest yield was obtained with 60% ethanol, at 3.26 ± 0.07 mg/g of dried powder, followed by 70%, 80%, 100%, 20%, and 40% ethanol, which yielded 2.92 ± 0.17, 2.84 ± 0.25, 2.66 ± 0.21, 1.65 ± 0.14, and 1.43 ± 0.09 mg/g, respectively. Water alone produced the lowest AA yield, at 0.92 ± 0.07 mg/g. Although AA exhibited the lowest Ra value with 100% ethanol (19.97), the highest extraction yield was obtained with 60% ethanol, which had an Ra value of 28.47 and a dielectric constant of 49.81. Thus, the extraction results did not correspond directly to the solvent system with the lowest Ra value.

Overall, 60% ethanol provided the greatest extraction efficiency for both AS and AA. This solvent system produced 9.94 ± 0.24 mg/g of AS and 3.26 ± 0.07 mg/g of AA. The results indicate that the combined assessment of Ra, dielectric constant, and experimental extraction yield is more appropriate than reliance on any single solvent parameter. In particular, an intermediate ethanol concentration appears to provide a suitable balance of solvent polarity and solute–solvent interactions for the recovery of the two major triterpenoids from dried *C. asiatica* leaves.

Accordingly, the 60% ethanol leaf extract was selected for further evaluation of its total phenolic content (TPC), total flavonoid content (TFC), antioxidant activity, neuroprotective activity, and anti-inflammatory mechanisms, including its effects on TNF-α, caspase-3, HO-1, and IL-18.

### 2.5. Assessing the Environmental Sustainability of the Extraction and Analytical Methods

**Figure 4:**
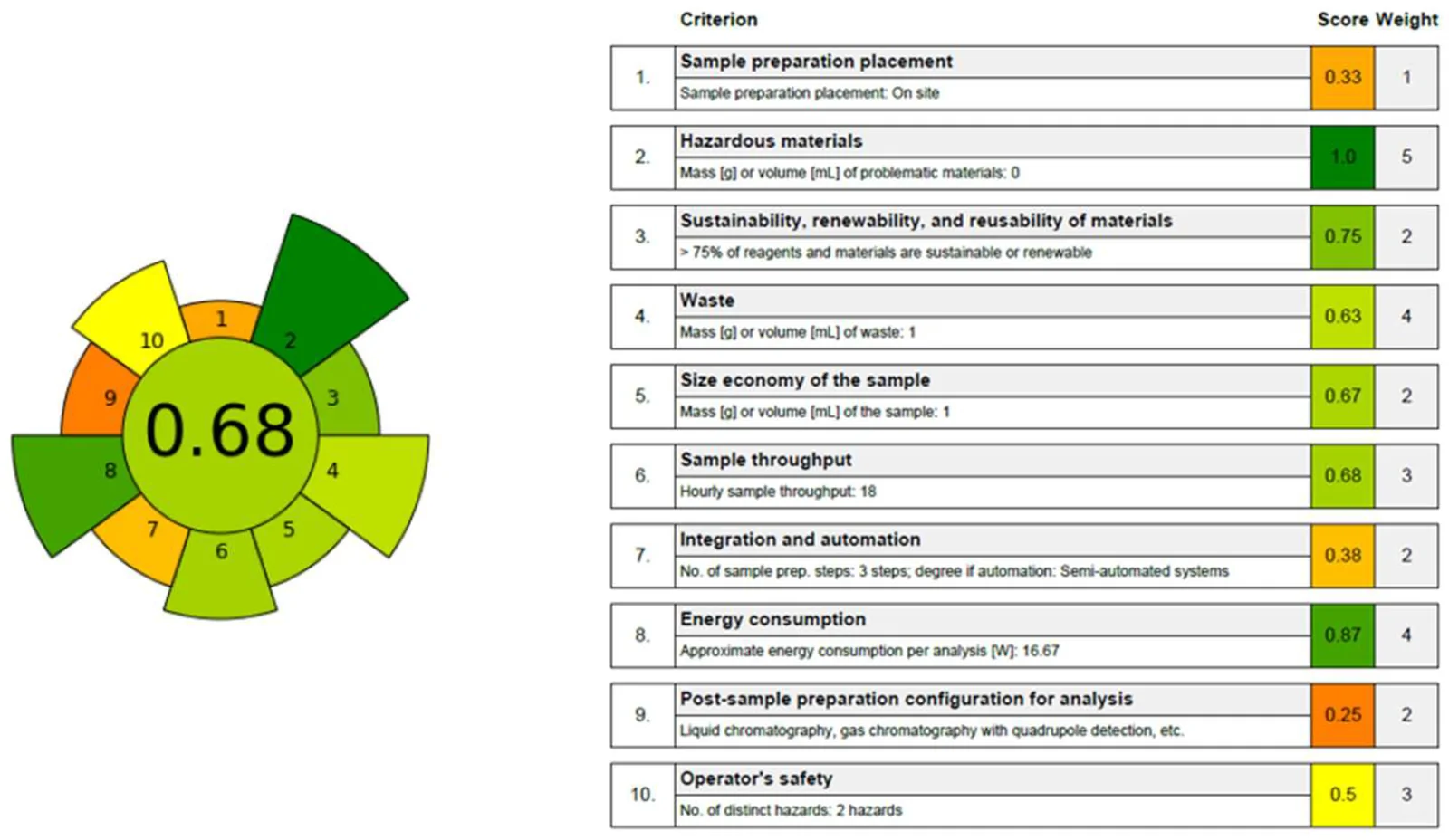
Assessment of the greenness of triterpenoid extraction and analysis methods using AGREEprep™ software (Web App Version 1.0). AGREEprep scores are shown on a sequential green-to-red color scale, ranging from green (score = 1.0) to red (score = 0.0).

The environmental sustainability of the sample-preparation strategies employed for the analysis of asiaticoside (AS) and asiatic acid (AA) in *C. asiatica* leaf extracts was evaluated using the AGREEprep metric, as described in Section 4.6. AGREEprep is an analytical tool that assesses the sustainability of sample-preparation procedures according to ten predefined criteria and a penalty-point system. The resulting score ranges from 0 to 1, with scores of 0 and 1 representing the lowest and highest levels of greenness, respectively. Thus, higher AGREEprep scores indicate more environmentally sustainable procedures [26,27]. Figure 4 illustrates the AGREEprep evaluation of the most promising extraction methods for AS and AA, as identified in Section 4.3 and Section 4.4. The ethanol–water extraction systems, together with the subsequent sample preparation and HPLC analysis of AS and AA in *C. asiatica* leaves, yielded a mean AGREEprep score of 0.68. Notably, the ethanol–water system eliminated the use of hazardous solvents. These findings highlight the important role of solvent selection in improving the environmental sustainability of AS and AA extraction procedures.

**Figure 5:**
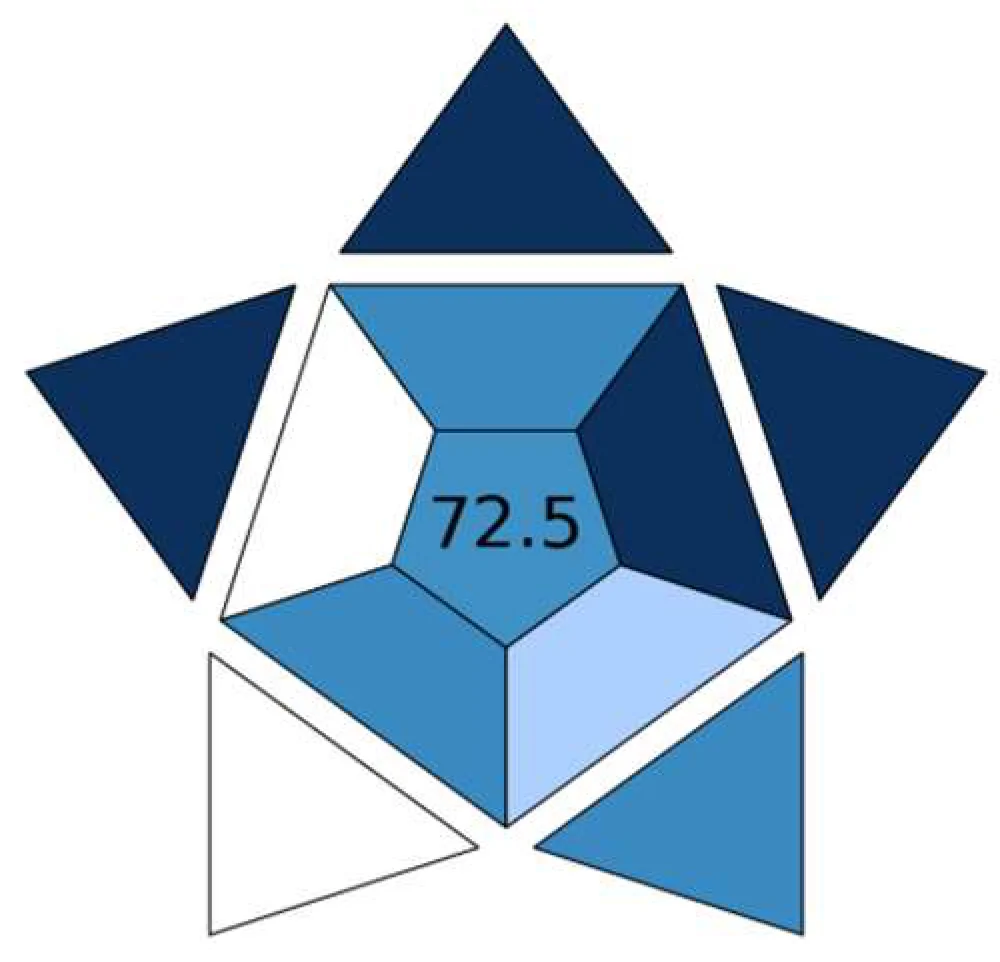
Assessment of the greenness of the analytical method for asiaticoside and asiatic acid using BAGI software (version 0.9.2). BAGI scores are shown on a sequential blue color scale, ranging from dark blue (score = 10) to white (score = 0).

The Blue Applicability Grade Index (BAGI) is a complementary metric used to evaluate the practical applicability, efficiency, speed, and cost-effectiveness of analytical methods. It assesses ten key characteristics of an analytical procedure: (1) type of analysis, (2) multi- or single-analyte analysis, (3) analytical technique, (4) simultaneous sample preparation, (5) sample-preparation requirements, (6) sample throughput, (7) reagents and materials, (8) preconcentration, (9) degree of automation, and (10) sample amount. The BAGI score ranges from 25 to 100, with higher scores indicating greater analytical applicability [28]. As shown in Figure 5, the extraction, sample preparation, and analysis procedures for asiaticoside (AS) and asiatic acid (AA) achieved a BAGI score of 72.5. This score indicates that the proposed method offers good practical applicability, efficiency, user-friendliness, and sustainability, making it suitable for routine extraction and analytical applications.

### 2.6. Total Phenolics, Total Flavonoids and Antioxidant Activities of Centella asiatica

The data presented in Table 8 provides a comprehensive assessment of the phenolic and flavonoid contents, as well as the antioxidant activity, of the 60% ethanol extract of *C. asiatica* leaves. The total phenolic content (TPC) was expressed as milligrams of gallic acid equivalents per gram of dried *C. asiatica* powder (mg GAE/g) and calculated using the calibration equation Y = 44.54X + 0.0251, with a coefficient of determination (R^2^) of 0.9982. The total flavonoid content (TFC) was expressed as milligrams of quercetin equivalents per gram of dried *C. asiatica* powder (mg QE/g) and determined using the calibration equation Y = 5.9051X + 0.0506, with an R^2^ value of 0.9901. The extract contained 7.61 ± 0.24 mg GAE/g of phenolic compounds and 22.64 ± 3.71 mg QE/g of flavonoids. These constituents may be associated with chlorogenic acid derivatives and flavonoid compounds naturally present in *C. asiatica* leaves [36].

The antioxidant activity of the extract and selected reference compounds was evaluated using the DPPH (2,2-diphenyl-1-picrylhydrazyl) radical-scavenging assay. Antioxidant activity was expressed as the half-maximal inhibitory concentration (IC_50_), defined as the concentration required to inhibit 50% of the DPPH radicals. The assay is based on the spectrophotometric measurement of the decrease in absorbance resulting from the reduction of DPPH radicals by electron- or hydrogen-donating compounds. Therefore, the observed response provides an indirect indication of the radical-scavenging and electron-donating capacities of the tested samples.

As shown in Table 8, the 60% ethanol extract exhibited substantial DPPH radical-scavenging activity, with an IC_50_ value of 0.12 ± 0.01 mg/mL. This activity may reflect the combined effects of the phenolic and flavonoid constituents extracted from the *C. asiatica* leaves. In contrast, purified AS and AA did not achieve 50% inhibition at the concentrations tested; consequently, their IC_50_ values could not be determined under the experimental conditions. These findings suggest that the antioxidant activity of the crude extract cannot be attributed solely to AS or AA and may instead result from the presence of other more reactive constituents, potential synergistic interactions, or both.

The relationship between frontier molecular orbital (FMO) properties and antioxidant activity was further examined to provide insight into the electronic characteristics and potential electron-transfer behavior of the tested compounds. The relevant parameters included the energies of the highest occupied molecular orbital (HOMO) and the lowest unoccupied molecular orbital (LUMO), together with the HOMO–LUMO energy gap (ΔE). In general, a smaller ΔE indicates that less energy is required for electronic excitation and may be associated with greater chemical reactivity and electron-transfer potential. However, ΔE alone does not fully determine antioxidant activity, which is also influenced by molecular structure, hydroxyl-group arrangement, resonance stabilization, and the extent of electronic delocalization.

As summarized in Table 8, AS exhibited the largest HOMO–LUMO gap, with a ΔE of 13.6957 eV, which is consistent with its weak DPPH radical-scavenging activity. Asiatic acid displayed a smaller ΔE of 9.5993 eV, comparable to that of quercetin, which had a ΔE of 9.9001 eV. Nevertheless, AA did not produce sufficient DPPH radical inhibition to permit calculation of its IC_50_, whereas quercetin exhibited potent antioxidant activity, with an IC_50_ value of 0.0018 ± 0.0001 mg/mL. This comparison demonstrates that the HOMO–LUMO gap should not be interpreted as an independent predictor of antioxidant capacity.

**Figure 6:**
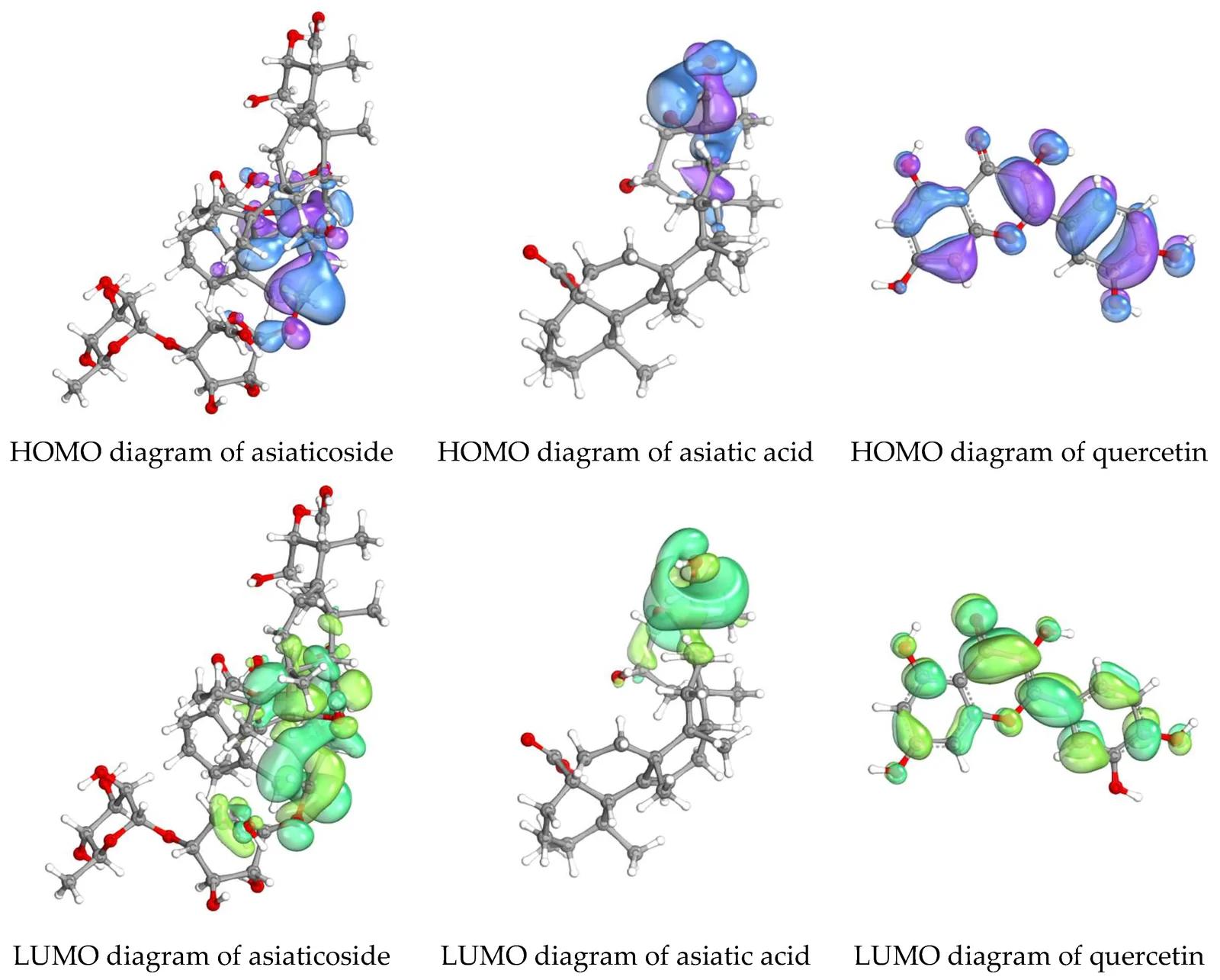
Frontier molecular orbital diagrams of asiaticoside, asiatic acid, and quercetin. The highest occupied molecular orbital (HOMO) is represented by violet and blue regions, whereas the lowest unoccupied molecular orbital (LUMO) is represented by light- and dark-green regions.

The strong antioxidant activity of quercetin may be attributed to its extended conjugated system and multiple hydroxyl groups. These structural features facilitate electron or hydrogen donation and enable stabilization of the resulting radical through resonance and electronic delocalization. As illustrated in Figure 6, the HOMO and LUMO of quercetin are broadly distributed across its molecular framework. In contrast, AS and AA possess less extensive conjugation, and their frontier molecular orbitals are localized in more limited regions of their structures. This restricted orbital delocalization may reduce their capacity to stabilize oxidized intermediates and may help explain their comparatively weak DPPH radical-scavenging activity. Overall, the results indicate that the antioxidant activity of the 60% ethanol extract is likely associated with its collective phytochemical composition rather than with the isolated triterpenoids alone.

### 2.7. Cytotoxicity and Neuroprotective Activity of Centella asiatica Leaf Extracts, Asiaticoside and Asiatic Acid

The cytotoxicity of the *C. asiatica* leaf extract, AS, and AA were evaluated in cultured SH-SY5Y neuroblastoma cells using the CCK-8 assay. This analysis was performed to assess cellular safety and to identify the highest non-toxic concentration of each test sample following a 24 h incubation period. Cell viability was expressed as a percentage relative to the untreated control group, as shown in Table 9.

The 60% ethanol extract of *C. asiatica* leaves exhibited its greatest cytotoxic effect at 1 mg/mL, at which cell viability decreased to 1.27 ± 3.97%. At 0.1 mg/mL, cell viability was reduced to 81.73 ± 2.90%. In contrast, concentrations of 0.01 mg/mL and lower did not produce apparent cytotoxicity, and cell viability remained close to 100%. Because the subsequent experiments were designed to investigate the neuroprotective activity of the extract, a concentration of 0.0001 mg/mL was selected. This concentration maintained cell viability above 100% and was therefore considered suitable for further testing.

AS did not exhibit detectable cytotoxicity toward SH-SY5Y cells at any of the tested concentrations, ranging from 0.0001 to 1 µM. For the subsequent neuroprotection experiments, a concentration of 0.01 µM was selected because it produced cell viability above 100% and was considered appropriate for further evaluation.

AA produced a clear cytotoxic effect at 1 nM, reducing cell viability to 74.92 ± 11.30%. However, cell viability increased as the concentration was reduced to the lower tested levels, ranging from 0.1 nM to 0.1 pM. A concentration of 10 pM was selected for the subsequent neuroprotective experiments because it maintained cell viability above 100% and was therefore considered non-toxic under the experimental conditions.

Overall, the CCK-8 assay indicated that the selected concentrations of the *C. asiatica* extract, AS, and AA were suitable for subsequent investigations of their neuroprotective effects in SH-SY5Y cells. The concentrations yielding cell viability above 100% were selected as experimental concentrations; however, this increase should be interpreted as a relative assay response compared with the control rather than as a direct measure of increased cell number.

Compounds intended for human use must demonstrate an acceptable safety profile and minimal toxicity. Therefore, the potential toxicity of AS and AA was evaluated in silico using ProTox-III. As shown in Table 10, the compounds were assigned to one of six toxicity classes, ranging from Class 1, which represents the highest toxicity (LD_50_ ≤ 5 mg/kg), to Class 6, which denotes the lowest toxicity or absence of predicted toxicity (LD_50_ > 5000 mg/kg) [37]. AS was classified as a Class 5 compound, with a predicted LD_50_ of 4000 mg/kg, whereas AA was assigned to Class 4, with a predicted LD_50_ of 2000 mg/kg. Neither compound showed predicted activity for hepatotoxicity, respiratory toxicity, or neurotoxicity.

**Figure 7:**
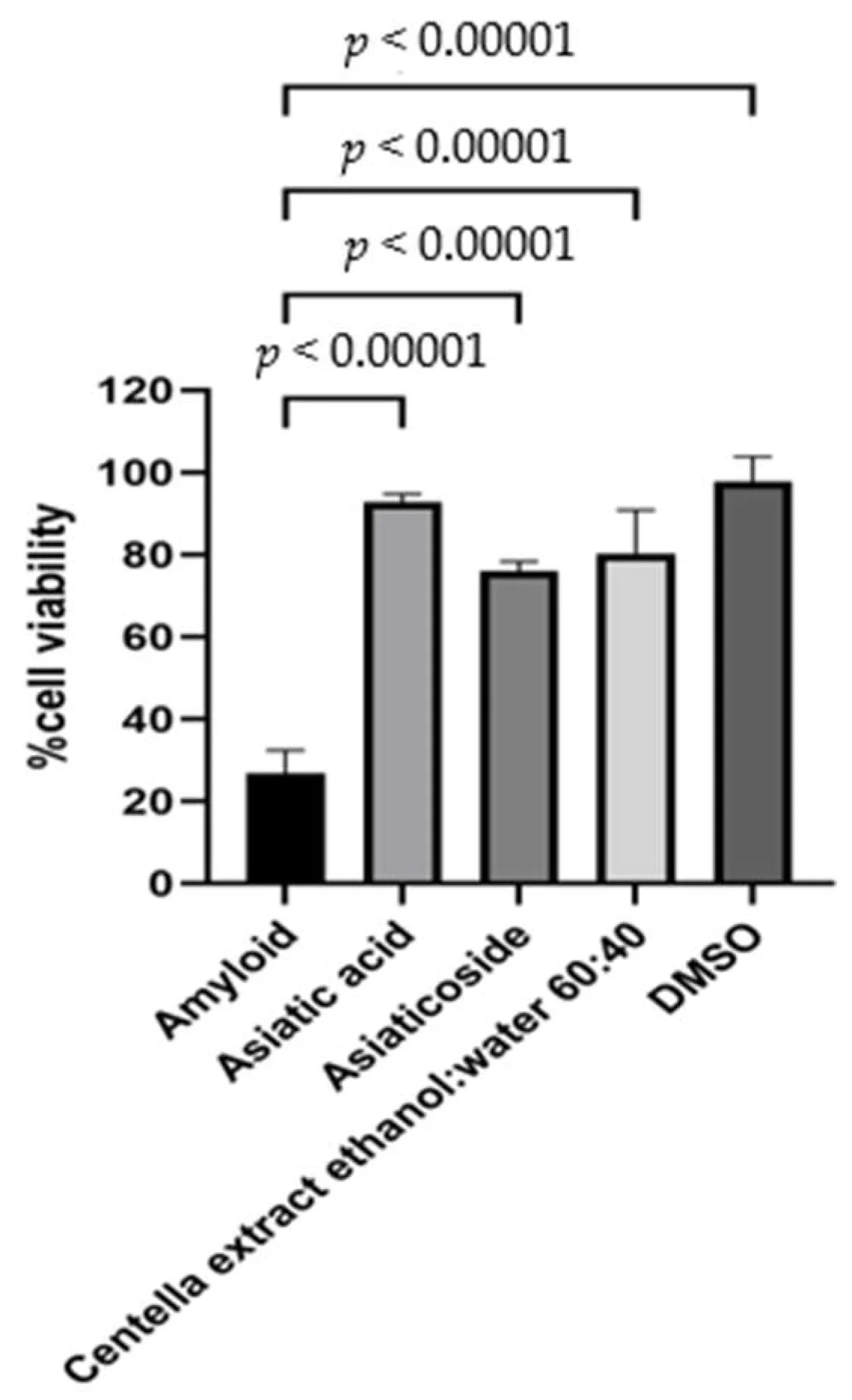
Neuroprotective effects of asiaticoside, asiatic acid, and *C. asiatica* leaf extract against 1.25 µM Aβ_42_-induced toxicity in SH-SY5Y cells.

The neuroprotective effects of the test compounds were evaluated in cultured SH-SY5Y neuroblastoma cells exposed to amyloid-β42 (Aβ_42_)-induced toxicity. Cell viability was compared among the DMSO-treated control group, the Aβ_42_-treated group, and groups co-treated with Aβ_42_ and either *C. asiatica* leaf extract (100 ng/mL), AA (10 pM), or AS (10 nM). The results, expressed as percentages of cell viability, are presented in Figure 7.

Exposure to Aβ_42_ alone markedly reduced cell viability to 26.84 ± 5.56%, compared with 97.81 ± 5.95% in the DMSO-treated control group. These findings confirm the cytotoxic effect of Aβ_42_ on SH-SY5Y neuroblastoma cells, while indicating that DMSO did not adversely affect cell viability under the experimental conditions.

Co-treatment with AA significantly increased cell viability to 92.81 ± 1.90% compared with the Aβ_42_-treated group (*p* < 0.0001). Similarly, AS co-treatment increased cell viability to 75.92 ± 2.43% (*p* < 0.0001), whereas co-treatment with *C. asiatica* leaf extract increased viability to 80.21 ± 10.62% (*p* < 0.0001).

Overall, all three treatments—AA, AS, and *C. asiatica* leaf extract—significantly attenuated Aβ_42_-induced cytotoxicity in SH-SY5Y cells. Among them, AA produced the greatest protective effect, restoring cell viability to a level comparable to that of the untreated control. These findings highlight the potential of AA as a promising neuroprotective compound against Aβ_42_-associated neuronal damage.

### 2.8. Effects of C. asiatica Leaf Extracts and Triterpenoids on Aβ_42_-Induced SH–SY5Y Cells

To compare the anti-inflammatory and neuroprotective effects of *C. asiatica* leaf extract with those of its individual triterpenoids, SH-SY5Y cells were stimulated with amyloid-β (Aβ_42_; 1.25 μM) and subsequently treated with AS (10 nM), AA (10 pM), or *C. asiatica* leaf extract (100 ng/mL). All treatments maintained cell viability above 80%, indicating that the tested concentrations were not cytotoxic to SH-SY5Y cells (Table 11). Compared with Aβ_42_ treatment alone, AS, AA, and *C. asiatica* extract significantly reduced Aβ_42_-induced TNF-α production from 1.55 ± 0.05 pg/mL to 0.39 ± 0.11, 0.67 ± 0.07, and 0.76 ± 0.02 pg/mL, respectively. A similar inhibitory pattern was observed for IL-18 production. Aβ_42_ exposure increased IL-18 levels to 4.40 ± 0.79 pg/mL, whereas treatment with AS, AA, and *C. asiatica* extract reduced IL-18 levels to 0.46 ± 0.17 pg/mL, 1.75 ± 0.60 pg/mL, and 0.56 ± 0.17 pg/mL, respectively. These findings indicate that all three treatments exerted significant anti-inflammatory effects by suppressing Aβ-induced TNF-α and IL-18 production (*p* ≤ 0.001).

Among the tested treatments, AA exhibited a comprehensive neuroprotective trend relative to the Aβ_42_-treated group. In addition to reducing the production of pro-inflammatory cytokines, AA significantly enhanced HO-1 production compared with the Aβ_42_-treated group, increasing its level from 10.80 ± 0.29 to 16.91 ± 2.19 pg/mL (*p* ≤ 0.001). Asiatic acid also significantly decreased the level of the apoptotic marker caspase-3 from 0.04 ± 0.01 to 0.02 ± 0.01 ng/mL (*p* < 0.05). In contrast, AS and *C. asiatica* extract did not significantly alter caspase-3 production compared with the Aβ_42_-treated group.

AA exhibited a broad protective effect against Aβ_42_-induced cellular injury in SH-SY5Y cells. Its activity was characterized by the simultaneous suppression of inflammatory cytokines, enhancement of the antioxidant defense marker HO-1, and inhibition of apoptosis, as indicated by reduced caspase-3 levels. Although AS and *C. asiatica* extract also significantly attenuated Aβ-induced inflammation, AA demonstrated the most comprehensive neuroprotective effect under the experimental conditions used. These findings suggest that AA may be a particularly promising candidate for further investigation as a neuroprotective compound.

### 2.9. Molecular Docking Analysis of Asiaticoside and Asiatic Acid with TNF-α, IL-18, HO-1, and Caspase-3

Given our observation that AS and AA inhibited the production of TNF-α, IL-18, and caspase-3, while AA stimulated HO-1 production, we further investigated the molecular interactions of these compounds with the corresponding target proteins. Molecular docking was performed using AutoDock 4.0 [38] to estimate the binding affinities of AS and AA toward TNF-α (PDB: 2AZ5 [39]), IL-18 (PDB: 3WO2 [40]), HO-1 (PDB: 3HOK [41]), and caspase-3 (PDB: 6CL0 [42]) and to compare their binding modes. This analysis was intended to provide insight into the predicted molecular mechanisms underlying their anti-inflammatory, anti-apoptotic, and antioxidant effects.

To validate the docking protocol and assess its ability to reproduce experimentally observed binding conformations, a redocking experiment was performed using the corresponding co-crystallized ligands. As shown in Table 9, the root-mean-square deviation (RMSD) values for the four redocked co-crystallized ligands from PDB structures 2AZ5, 3WO2, 3HOK, and 6CL0 were 1.858 Å, 1.591 Å, 1.667 Å, and 1.514 Å, respectively. All values were ≤2.0 Å, indicating that the docking parameters and protocol reliably reproduced the native binding poses.

As shown in Table 12, the binding free energy (ΔG) values were generally consistent with the predicted inhibition constants (Kᵢ), with more negative ΔG values corresponding to lower Kᵢ values. AA exhibited more favorable binding energies and lower predicted Kᵢ values than AS for all four target proteins. These findings suggest that AA has a higher predicted binding affinity for TNF-α, IL-18, HO-1, and caspase-3 than AS. The comparatively weaker binding affinity of AS may be attributable to its larger molecular size and greater hydrophilicity, which may limit its compatibility with the predominantly hydrophobic regions of the active sites of these target proteins.

**Figure 8:**
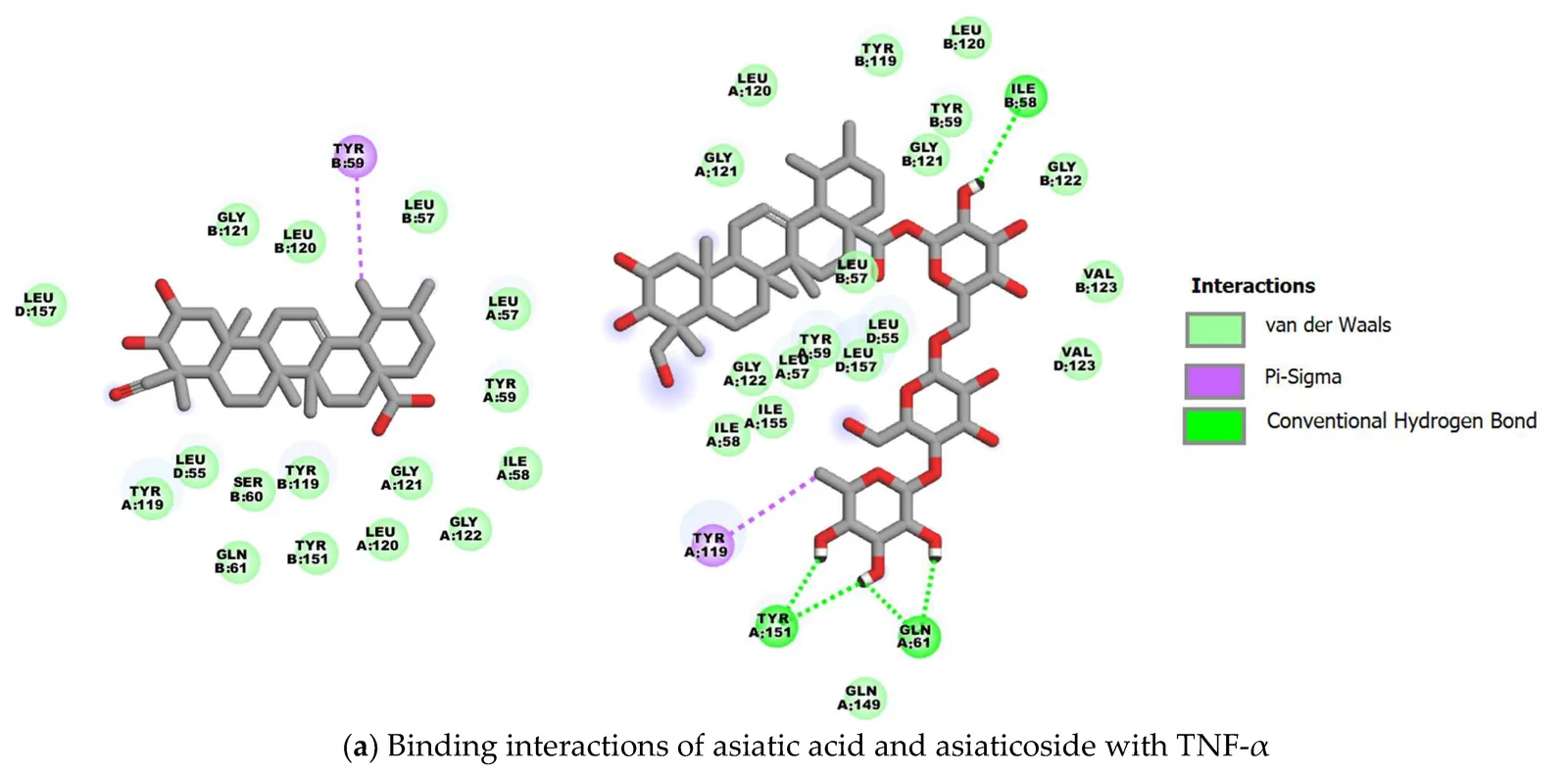

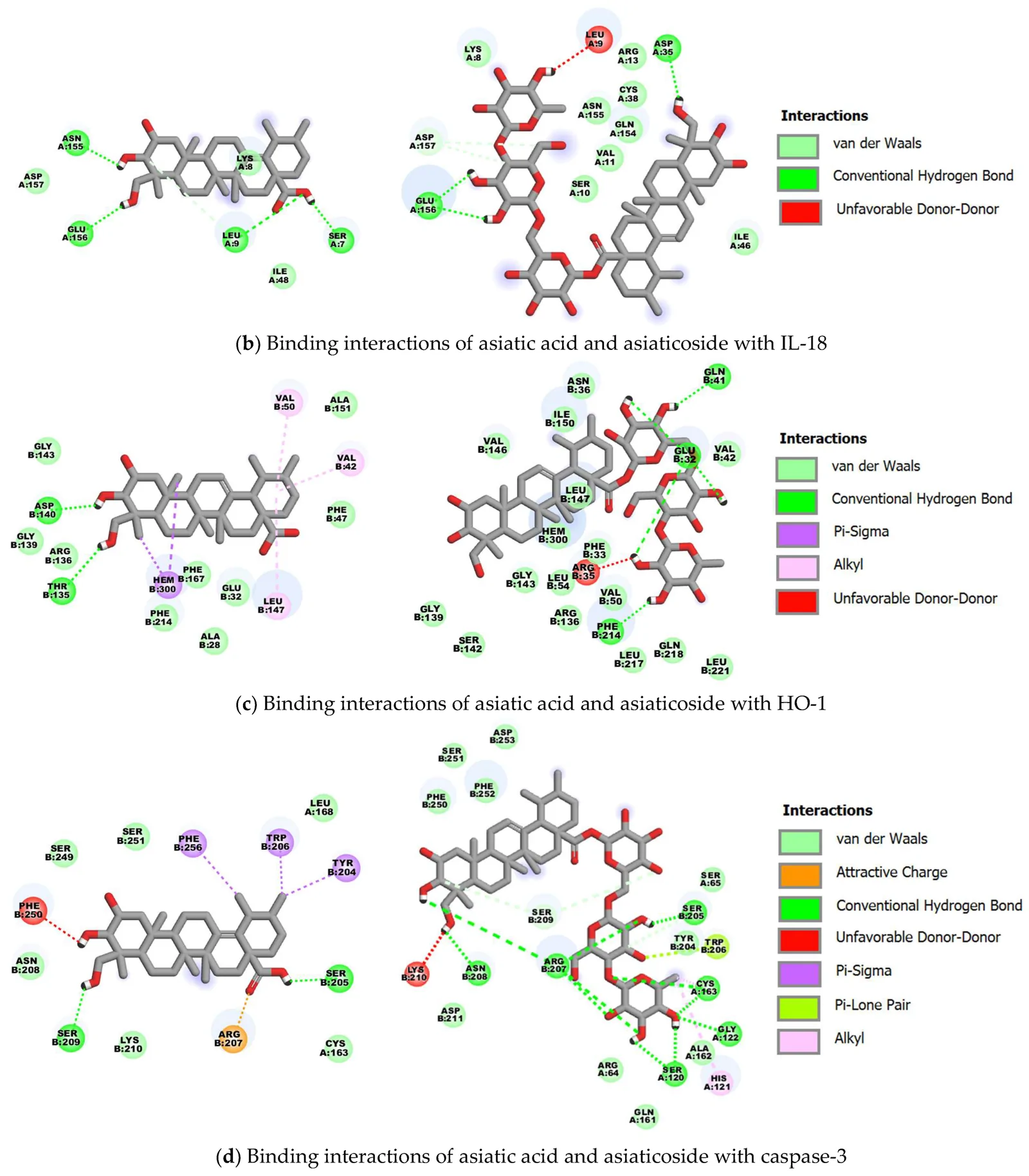
Two-dimensional representations of the binding interactions of asiaticoside and asiatic acid with (**a**) TNF-α, (**b**) IL-18, (**c**) HO-1, and (**d**) caspase-3. Dashed lines represent specific types of noncovalent intermolecular bonds or interactions between a ligand and the amino acid residues of a macromolecule.

As shown in Figure 8, unfavorable donor–donor interactions were observed in the AS and AA complexes with IL-18 (Leu9), HO-1 (Arg35), and caspase-3 (Lys210, Phe250). These interactions may have contributed to the differences in the predicted ΔG values between the two compounds. For IL-18, the ΔG values were −1.16 kcal/mol for AS and −4.02 kcal/mol for AA. For HO-1, the corresponding values were −4.41 and −9.20 kcal/mol, respectively, whereas for caspase-3, they were −6.83 and −7.95 kcal/mol, respectively. In the TNF-α complexes, AA exhibited a slightly more favorable binding energy than AS, with ΔG values of −8.60 and −8.08 kcal/mol, respectively. Neither compound formed unfavorable donor–donor interactions with TNF-α.

The binding interactions between the ligands and their respective protein targets were analyzed using Discovery Studio Visualizer. As shown in Figure 8, AS formed hydrogen-bond interactions with Gly61 and Tyr151 in chain A and with Leu58 in chain B of TNF-α. In contrast, no hydrogen-bond interactions were observed between AA and TNF-α. The favorable interactions observed for AA with TNF-α may be attributed to its hydrophobic triterpenoid nucleus. The amino acid residues involved in the interactions with each ligand are summarized in Table 12.

## 3. Discussion

The present study highlights the value of integrating HSPs, green extraction methods, chemical characterization, and complementary in vitro and in silico approaches to investigate the neuroprotective potential of *C. asiatica* triterpenoids. AS and AA exhibit markedly distinct structural and physicochemical properties. The trisaccharide moiety of AS increases its molecular size, hydrogen-bonding capacity, polarity, and hydrophilicity, whereas AA has a comparatively less polar triterpenoid structure. These differences were consistent with the SwissADME predictions [32]. Although AS is expected to be more water-compatible than AA, its high molecular weight, greater numbers of hydrogen-bond donors and acceptors, and increased topological polar surface area may limit passive gastrointestinal permeability. AA also exhibited low predicted gastrointestinal absorption, suggesting that both compounds may require formulation-based approaches or alternative delivery routes to enhance systemic exposure.

These predictions are consistent with the pharmacokinetic findings [43]. Following oral administration, AS was rapidly absorbed, reaching peak plasma concentrations within 5–15 min; however, its oral bioavailability was less than 1%. Within 1 h of administration, AS was extensively distributed to the brain, stomach, and skin and remained detectable in these tissues for at least 4 h. After injection, AS was excreted predominantly in its unchanged form, whereas oral administration resulted mainly in the fecal excretion of AA metabolites [44]. Collectively, these findings indicate that the structural properties of AS and AA strongly influence their absorption, distribution, and elimination profiles and should therefore be considered when designing delivery systems intended to improve their neuroprotective efficacy.

HPLC–PDA analysis confirmed the presence of asiaticoside (AS) and asiatic acid (AA) in the *C. asiatica* leaf extracts. AS eluted before AA, consistent with its greater polarity and weaker retention under the chromatographic conditions. The extraction results further demonstrated that solvent composition substantially influenced the recovery of both triterpenoids. Hansen solubility parameter (HSP) analysis predicted that AS would interact favorably with intermediate ethanol–water mixtures, whereas AA would exhibit a greater affinity for ethanol [23]. However, the lowest HSP distance (Ra) did not directly correspond to the highest experimental extraction yield. Among the tested solvent systems, 60% ethanol produced the highest recoveries of both AS and AA, yielding 9.94 ± 0.24 and 3.26 ± 0.07 mg/g of dried plant material, respectively. Although AS and AA exhibit markedly different octanol–water partition coefficients (log P = −0.33 and 4.45, respectively), 60% ethanol served as the optimal solvent system for both compounds. This demonstrates that log P alone is not the sole determining factor for extraction efficiency. Instead, extraction yield is collectively governed by Hansen solubility parameters (HSPs), dielectric constant, solvent penetration, mass transfer, and plant cell wall matrix swelling [13]. While the ethanol fraction offers suitable lipophilicity to interact with the triterpenoid backbone, the aqueous fraction lowers interfacial tension and promotes matrix swelling, thereby facilitating solvent penetration. The superior performance of 60% ethanol is thus attributed to its optimal balance of polar and less-polar interactions, enabling the efficient simultaneous recovery of both the polar glycoside AS and the less-polar aglycone AA. The superior performance of 60% ethanol may be attributed to its optimal balance of polar and less-polar interactions, which facilitates the simultaneous extraction of glycosylated AS and the less-polar aglycone AA.

In the present study, dried *C. asiatica* leaf powder was extracted with 60% ethanol at a solid-to-liquid ratio of 1:10 (*w*/*v*), using sonication for 30 min at 40 kHz and 300 W. By comparison, Seong et al. (2021) reported an AS yield of 19.71 mg/g dry weight using 80% methanol, sonication for 30 min, and an applied power of 200 W [44]. The higher AS yield reported in that study may be attributable to differences in the botanical source and plant material, as well as to variations in solvent composition and extraction conditions. Methanol has a smaller molecular size and generally higher polarity than ethanol, which may enhance its penetration into plant tissues and improve the solubilization of polar glycosylated triterpenoids. Nevertheless, methanol is less desirable from a green-extraction perspective because of its higher toxicity, whereas ethanol is a safer and more environmentally compatible solvent for the extraction of bioactive compounds.

**Figure 9:**
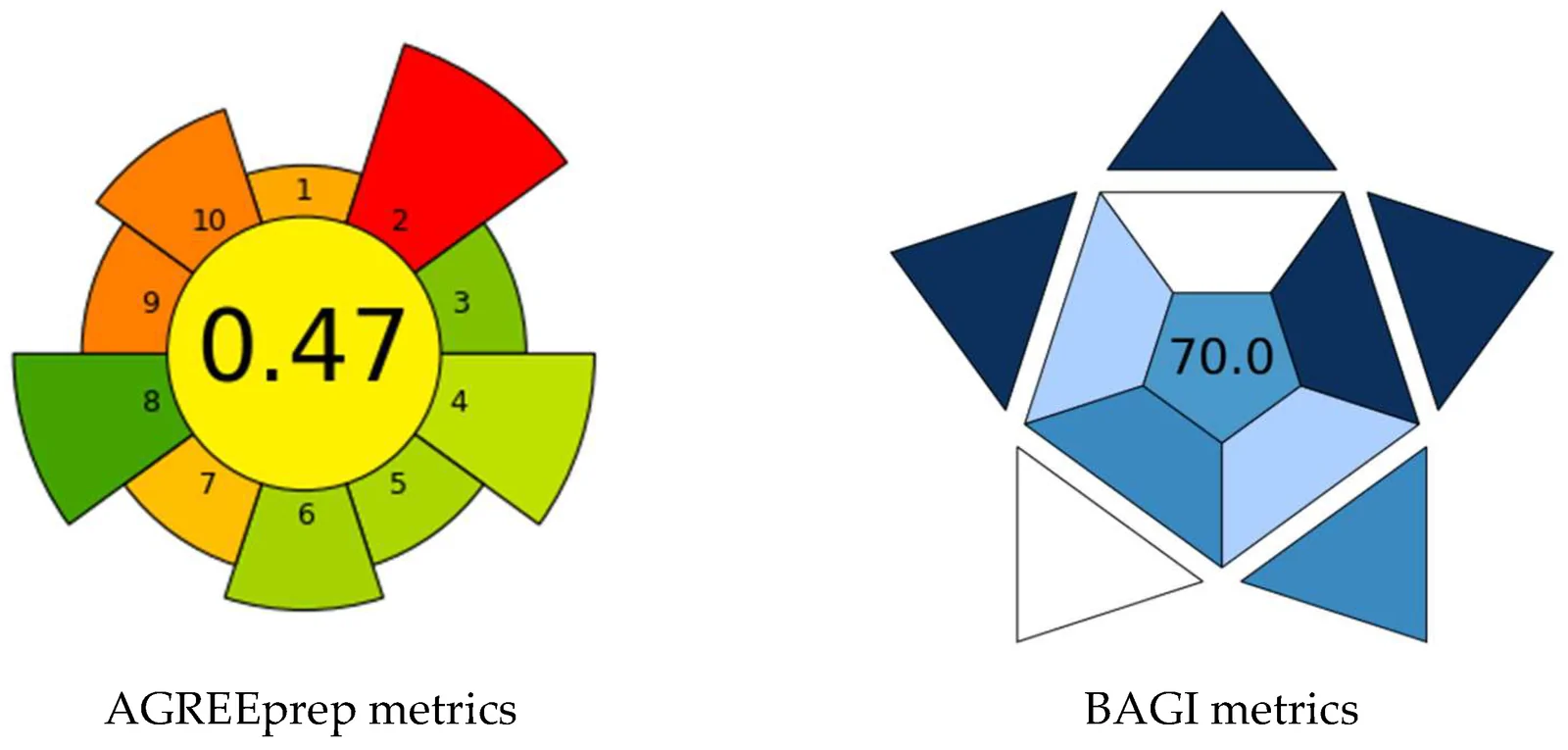
Assessment of the greenness of the triterpenoid extraction and analysis methods using the AGREEprep and BAGI metrics for a hydro-methanolic extraction solvent system. AGREEprep scores are shown on a sequential green-to-red color scale, ranging from green (score = 1.0) to red (score = 0.0), while BAGI scores are shown on a sequential blue color scale, ranging from dark blue (score = 10) to white (score = 0).

The proposed extraction procedure demonstrated favorable environmental performance and practical applicability. Its AGREEprep score of 0.68 indicates a comparatively high degree of greenness, largely attributable to the use of ethanol and water as alternatives to more hazardous organic solvents (Figure 4) [26,27]. In addition, the BAGI score of 72.5 supports the method’s suitability for routine analysis, reflecting its relatively simple sample preparation, simultaneous determination of two analytes, and satisfactory analytical efficiency (Figure 5) [28,29]. In comparison, the hydro-methanolic extraction method, considered a less environmentally preferable alternative, produced lower AGREEprep and BAGI scores of 0.47 and 70.0, respectively (Figure 9). The higher AGREEprep score obtained for the ethanol–water system suggests reduced environmental and health concerns associated with solvent selection. Ethanol is generally less toxic and more readily acceptable for sustainable extraction than methanol, while also offering practical advantages related to handling and solvent disposal.

Nevertheless, these greenness metrics primarily assess the analytical workflow and should not be interpreted as a complete evaluation of process sustainability. A comprehensive assessment should also consider energy consumption during extraction and solvent evaporation, solvent recovery and recycling, waste generation, material use, worker safety, and the feasibility of process scale-up. Therefore, although the ethanol–water system represents a more sustainable alternative to the hydro-methanolic system, further evaluation under pilot- or industrial-scale conditions is required to confirm its overall environmental advantages.

The 60% ethanol extract contained appreciable phenolic and flavonoid levels and showed strong DPPH radical-scavenging activity. In contrast, isolated AS and AA did not reach the DPPH IC_50_ under the tested conditions. Therefore, the antioxidant activity of the extract is unlikely to result primarily from these triterpenoids and may instead reflect phenolic constituents, flavonoids, or synergistic interactions among multiple compounds. The frontier molecular orbital results support this interpretation. Although AA had a smaller HOMO–LUMO gap than AS, it was not strongly active in the DPPH assay [30,31]. Thus, ΔE alone cannot predict antioxidant capacity, which depends additionally on conjugation, hydroxyl-group arrangement, resonance stabilization, and radical-scavenging mechanisms [24].

Importantly, the biological assays identified AA as the most promising neuroprotective constituent. At non-cytotoxic concentrations, AA, AS, and the crude extract significantly improved SH-SY5Y viability following Aβ_42_ exposure [44]. AA produced the greatest recovery, restoring viability close to the untreated control. Its broader activity was accompanied by suppression of TNF-α and IL-18, enhancement of HO-1, and reduction of caspase-3. AS and the extract also reduced inflammatory cytokines, suggesting that both individual constituents and phytochemical combinations contribute to neuroprotection. In the cellular mechanism evaluation, AS (10 nM), AA (10 pM), and the 60% ethanol extract (100 ng/mL) were evaluated at single, individually optimized concentrations. While this screening design successfully demonstrated cytoprotective and anti-inflammatory activity, it reflects compound efficacy solely at the chosen working concentrations. Because the tested concentrations span different orders of magnitude, relative pharmacological potency cannot be inferred from these single-concentration comparisons. Establishing full concentration–response curves and determining exact potency metrics remain an important direction for future mechanistic investigations.

ProTox-III predicted relatively low acute toxicity for both compounds, with no hepatotoxic, respiratory, or neurotoxic activities. However, the computational tool flagged potential cardiotoxicity and nephrotoxicity endpoints for both AS and AA (Table 10). While these in silico predictions provide useful initial screening flags, computational models rely on structural pharmacophores that can generate false positives without accounting for physiological metabolism, active transport, or tissue exposure dynamics. Given the established safety history of *C. asiatica*, these predicted organ toxicity endpoints should be interpreted with caution, and targeted in vitro and in vivo toxicological studies remain necessary to confirm their safety profiles [37]. Furthermore, biological evaluation in this study was restricted to the SH-SY5Y cell line. While unstimulated cells exhibited no loss of viability across the working concentrations, the absence of comparative toxicity testing in non-cancerous normal cell lines (e.g., HEK293 or L-02) limits our ability to determine the exact selectivity index and constitutes a study limitation.

The molecular docking results were consistent with the observed cellular responses. AA exhibited more favorable predicted binding energies and lower inhibition constants than AS when interacting with TNF-α, IL-18, HO-1, and caspase-3, suggesting a comparatively stronger binding affinity for these target proteins. This difference may be related to the structural characteristics of the two compounds. AA has a smaller, less hydrophilic molecular framework that may facilitate deeper penetration into binding pockets and promote more effective interactions with hydrophobic amino acid residues. In contrast, the larger and more hydrophilic structure of AS, which contains a glucose moiety, may impose steric and conformational constraints and reduce its compatibility with the predominantly hydrophobic regions of these active or ligand-binding sites [6].

The stronger predicted interactions of AA provide a plausible molecular basis for its greater anti-inflammatory, antioxidant-defense, and anti-apoptotic activities observed at the cellular level. Nevertheless, these findings should be interpreted cautiously because molecular docking is based on a relatively static representation of ligand–protein interactions and does not demonstrate direct target engagement, binding under physiological conditions, or a causal pharmacological relationship [45]. Further validation using biochemical binding assays, enzymatic inhibition studies, and molecular-dynamics simulations is therefore necessary to confirm the proposed interactions.

Among the tested constituents, AA exhibited the most pronounced neuroprotective activity in Aβ_42_-exposed SH-SY5Y cells. Its effects were associated with improved cell viability, suppression of the pro-inflammatory mediators TNF-α and IL-18, enhancement of the cytoprotective enzyme HO-1, and reduction of caspase-3, suggesting the simultaneous attenuation of neuroinflammation, oxidative stress, and apoptosis. Although AS and the crude extract also demonstrated protective effects, the stronger activity of AA may be related to its smaller and less hydrophilic structure, which could facilitate more favorable interactions with relevant protein targets.

The antioxidant potential observed in this study was assessed using the DPPH radical-scavenging assay. Since this assay measures a specific aspect of antioxidant capacity, the results should be interpreted as evidence of DPPH radical-scavenging activity rather than comprehensive antioxidant activity. Future studies using complementary assays, such as ABTS, FRAP, or ORAC, are needed to provide a broader evaluation.

Future studies should optimize extraction through response-surface methodology and assess solvent recycling and pilot-scale feasibility. Pharmacokinetic investigations are needed to clarify absorption, metabolism, tissue distribution, and brain penetration, particularly because both compounds showed poor predicted gastrointestinal absorption. Nanoformulations, phospholipid complexes, glycosylation strategies, or intranasal delivery could be explored to improve bioavailability. Mechanistic work should include pathway-focused experiments involving NF-κB, Nrf2/HO-1, inflammasome signaling, mitochondrial function, oxidative stress, and apoptosis. Finally, activity-guided fractionation, quantitative metabolomics, three-dimensional neuronal models, primary neurons, and animal models should be used to distinguish the effects of AA, AS, and extract-level synergy and to confirm efficacy and safety before clinical translation.

## 4. Materials and Methods

### 4.1. Materials

Plant material was collected from the Botanical Garden of the Faculty of Pharmacy at Srinakharinwirot University in Nakhon Nayok, Thailand. Ethanol, along with chromatography-grade acetonitrile and methanol, was purchased from Merck (Darmstadt, Germany), while formic acid was obtained from Sigma-Aldrich Co. (St. Louis, MO, USA). All reference standards were provided by ChemFaces (Wuhan, Hubei, China).

### 4.2. Calculation of Hansen Solubility Parameters

The Hansen solubility parameters (HSPs) of the triterpenoids were estimated from their molecular structures using the group contribution method proposed by Stefanis et al. [23]. The dispersion (δd), dipolar intermolecular (δp), and hydrogen-bonding (δh) components were determined using Equations (1)–(3), respectively.

(1) δd=∑iNiCi+W∑jMjDj+17.3231 MPa1/2

(2) $$\delta_{p}=\left(\sum_{i}N_{i}C_{i}+W\sum_{j}M_{j}D_{j}+7.3548\right){\mathrm{MPa}}^{1/2}$$

(3) $$\delta_{h}=\left(\sum_{i}N_{i}C_{i}+W\sum_{j}M_{j}D_{j}W+7.9793\right){\mathrm{MPa}}^{1/2}$$

In these equations, Ci represents the contribution of a first-order group of type i, which occurs Ni times in the molecule, whereas Dj denotes the contribution of a second-order group of type j, occurring Mj times. The binary constant W is assigned a value of 0 when no second-order groups are present and 1 when such groups occur. Solvents with HSP components most closely matching those of the flavonoids were selected for extraction. Solvent–solute compatibility was evaluated using the interaction radius (Ra), calculated according to Equation (4).

(4) $$R_{a}^{2}=4{\left(\delta_{\mathrm{di}}-\delta_{\mathrm{dj}}\right)}^{2}+{\left(\delta_{\mathrm{pi}}-\delta_{\mathrm{pj}}\right)}^{2}+{\left(\delta_{\mathrm{hi}}-\delta_{\mathrm{hj}}\right)}^{2}$$

where i refers to the HSPs of the solute, and j refers to the HSPs of the solvent.

### 4.3. Preparation of Herbal Extract

The cleaned leaves of *C. asiatica* were dried in a hot-air oven at 50 °C under atmospheric pressure for 24 h. The dried material was then finely pulverized using an herbal grinder and passed through a 40-mesh sieve. Extractions were carried out using ethanol, water, and a water–ethanol mixture as solvents. For each extraction, 1 g of the powdered sample was combined with the respective solvent at a solid-to-liquid ratio of 1:10 (*w*/*v*) and sonicated for 30 min at a frequency of 40 kHz and power of 300 W. The resulting mixtures were filtered through filter paper under vacuum, and the filtrates were concentrated using a rotary evaporator at a temperature below 70 °C. The crude extracts were collected and stored at 2–8 °C until further analysis.

### 4.4. HPLC-UV Analysis

High-performance liquid chromatography (HPLC) analyses were performed using an Agilent 1260 Infinity II system (Santa Clara, CA, USA) equipped with a quaternary pump, autosampler, column thermostat, and photodiode array detector. Chromatographic separation was achieved using an ACE C18-AR column (4.6 × 250 mm, 5 μm; Avantor, Aberdeen, UK) protected by a Phenomenex C18 guard column (4 × 3 mm, 5 μm; Torrance, CA, USA). The mobile phase consisted of ultrapure water (solvent A) and acetonitrile (solvent B). The elution program began with isocratic conditions at 25% solvent B for 10 min, followed by a linear gradient from 25% to 80% solvent B over 15 min (10–25 min). The mobile phase was then maintained at 80% solvent B for 5 min, decreased to 25% solvent B over 3 min, and held at 25% solvent B for a further 7 min to re-equilibrate the column. The mobile phase was delivered at a flow rate of 1.0 mL/min, while the column was maintained at 25 °C. A 10 µL volume of each sample was injected. The photodiode array (PDA) detector recorded absorbance across the wavelength range of 190–400 nm, with quantification performed at 210 nm. The analytical method was evaluated through system suitability testing and validated in accordance with the requirements of the International Council for Harmonisation (ICH) [34] and the AOAC Guidelines (2016) [35].

### 4.5. Method Validation

The HPLC–PDA method was validated according to the guidelines established by the International Council for Harmonisation (ICH) [34] and the Association of Official Analytical Collaboration (AOAC) [35]. The validation parameters included linearity, limit of detection (LOD), limit of quantification (LOQ), accuracy, and precision.

#### 4.5.1. Specificity

The specificity of the HPLC–PDA method was evaluated by comparing the chromatograms of the mixed reference standards with those of the sample extracts at 210 nm. The target compounds were identified by their retention times, and their peaks were confirmed to be baseline-resolved from neighboring chromatographic signals. Furthermore, specificity was verified through triplicate analyses of both the reference standards and sample extracts.

#### 4.5.2. Linearity

Five-point calibration curves were prepared for each reference standard over the concentration range of 32–180 µg/mL for AA and 32–1290 µg/mL for AS. The curves demonstrated excellent linearity, with correlation coefficients (r) exceeding 0.995, as required by AOAC guidelines. All measurements were conducted in triplicate.

#### 4.5.3. Limit of Detection (LOD) and Limit of Quantification (LOQ)

The limits of detection (LOD) and quantification (LOQ) were determined from the linear regression data using the following equations:

LOD = 3.3 × (standard deviation of the y-intercept/mean slope)

LOQ = 10 × (standard deviation of the y-intercept/mean slope)

Accordingly, the LOD and LOQ were calculated by multiplying the ratio of the standard deviation of the calibration-curve intercept to the mean slope by factors of 3.3 and 10, respectively.

#### 4.5.4. Accuracy

Method recovery was assessed by fortifying the samples at three concentration levels: 350, 500, and 650 µg/mL for AS, and 60, 90, and 120 µg/mL for AA. Recovery percentages were calculated by comparing the measured concentrations with the corresponding nominal spiked concentrations. Accuracy was expressed as the mean recovery (%) obtained from triplicate injections at the low, medium, and high concentration levels.

#### 4.5.5. Precision

Method precision was evaluated through repeatability and intermediate precision. Repeatability was assessed using three consecutive injections performed on the same day, whereas intermediate precision was determined from triplicate injections at low, medium, and high concentration levels over three different days. Precision was reported as the percentage relative standard deviation (%RSD).

### 4.6. Sustainability Assessment of the Extraction Methods

The environmental performance of the proposed extraction methods was evaluated using AGREEprep™ (Web App Version 1.0), an open-access software tool (https://agreeprep.anvil.app/ (accessed on 22 May 2026)) developed by Wojnowski et al. (2022) [26]. This tool is designed to assess the ecological impact of sample-preparation procedures based on ten principles of green sample preparation (GSP) proposed by Pena-Pereira et al. (2022) [26]. It evaluates ten environmental-impact categories and converts the results into normalized subscores ranging from 0 to 1. These subscores are then integrated into an overall score, where a value of 1 indicates the most environmentally sustainable procedure and 0 represents the least favorable performance. The results are displayed graphically as a pictogram.

The applicability of the analytical method was assessed using the Blue Applicability Grade Index (BAGI) metric (version 0.9.2) and its associated open-access software (https://bagi-index.anvil.app/ (accessed on 22 May 2026)) developed by Manousi et al. (2023) [28]. The BAGI assessment considers ten characteristics of an analytical procedure: (1) the type of analysis, (2) single- or multi-analyte determination, (3) analytical technique, (4) simultaneous sample preparation, (5) sample-preparation requirements, (6) sample throughput, (7) reagents and materials used, (8) preconcentration procedures, (9) degree of automation, and (10) sample amount.

### 4.7. Determination of Total Phenolic Content

Total phenolic content (TPC) was quantified using the Folin–Ciocalteu assay, with minor modifications to accommodate the sample matrix. Briefly, 1 mL of Folin–Ciocalteu reagent was combined with the sample extract at a concentration of 300 µg/mL and allowed to react for 5 min. Subsequently, 1 mL of 75 mg/mL sodium carbonate (Na_2_CO_3_) solution was added, and the mixture was diluted to a final volume of 10 mL with distilled water. The reaction mixture was incubated at room temperature for 90 min to allow complete color development. Absorbance was then measured at 765 nm against a reagent blank. A gallic acid calibration curve was prepared over the concentration range of 10–40 µM, and the TPC was reported as milligrams of gallic acid equivalents (GAE) per gram of extract. All measurements were performed in triplicate to ensure analytical reliability.

### 4.8. Determination of Total Flavonoid Content

Total flavonoid content (TFC) was determined using a modified aluminum chloride colorimetric assay. Briefly, 1 mL of 8 mg/mL aluminum chloride (AlCl_3_) solution was mixed with the sample extract at a concentration of 300 µg/mL and vortexed to achieve homogeneity. The mixture was incubated for 1 h, after which its absorbance was measured at 415 nm against a reagent blank. Quantification was based on a calibration curve prepared with quercetin standards over the concentration range of 15–80 µM. The results were expressed as milligrams of quercetin equivalents (QE) per milligram of extract. Each analysis was performed in triplicate to ensure the reliability and reproducibility of the results.

### 4.9. Evaluation of Antioxidant Activity

Antioxidant activity was initially assessed using the DPPH (2,2-diphenyl-1-picrylhydrazyl) radical-scavenging assay, with modifications based on a previously reported procedure. Briefly, 100 µL of the extract solution was mixed with 100 µL of 0.6 mM DPPH prepared in ethanol and vortexed thoroughly to ensure adequate contact between the reactants. The mixture was incubated at room temperature for 30 min in the dark. After incubation, absorbance was measured at 520 nm using a spectrophotometer. A control containing the same concentration of DPPH without the extract was prepared under identical conditions. Radical-scavenging activity (RSA) was expressed as the percentage of DPPH discoloration and calculated using the following equation:

RSA (%) = [(Acontrol − Asample)/Acontrol] × 100%(5)

where Acontrol denotes the absorbance of the DPPH solution prepared in methanol, while Asample represents the absorbance measured after mixing the DPPH solution with the plant extract. The IC_50_ values, which reflect antioxidant capacity, were estimated using linear regression analysis. Each sample was analyzed in triplicate to ensure the reliability and accuracy of the results.

### 4.10. Evaluation of Neurotoxicity and Neuroprotective Activity

#### 4.10.1. Cell Culture

SH-SY5Y cells (ATCC^®^ CRL-2266™) were purchased from American Type Culture Collection (Manassas, VA, USA) and cultured in a 1:1 (*v*/*v*) mixture of minimum essential medium (MEM; Gibco, Grand Island, NY, USA) and Ham’s F-12 nutrient mixture (F-12; Gibco, USA). The culture medium was supplemented with 10% (*v*/*v*) fetal bovine serum (FBS; Sigma-Aldrich), 1 mM sodium pyruvate (Gibco, USA), and 1% (*v*/*v*) antibiotic–antimycotic solution (Gibco, USA). Cells were maintained at 37 °C in a humidified atmosphere containing 5% CO_2_, following the method described previously [46].

#### 4.10.2. Determination of Neurotoxicity of *C. asiatica* Extract, Asiaticoside, and Asiatic Acid

SH-SY5Y cells were seeded at a density of 1 × 10^4^ cells per well in complete medium consisting of MEM and F-12 in a 1:1 (*v*/*v*) ratio, supplemented with 10% (*v*/*v*) FBS, 1 mM sodium pyruvate, and 1% (*v*/*v*) antibiotic–antimycotic solution. Afterward, the cells were treated with the 60% ethanol extract of *C. asiatica*, AS, or AA for 24 h. Untreated cells maintained in complete medium served as the experimental baseline. Cell viability was assessed using the CCK-8 assay. Following a 2 h incubation with the CCK-8 reagent, absorbance was recorded at 450 nm using a microplate spectrophotometer. Cells treated with 0.5% (*v*/*v*) DMSO in complete medium were used as the vehicle control. Three independent experiments were conducted, with each experiment performed in triplicate. Cell viability was calculated using the following equation:

% cell viability = OD of treated cell × 100/OD of untreated cell

#### 4.10.3. Neuroprotective Effect of *C. asiatica* Extract, Asiaticoside, and Asiatic Acid on Aβ_42_–Induced Toxicity in SH–SY5Y Cells

The neuroprotective activity of the test samples was evaluated using an amyloid β42 (Aβ_42_)-induced SH-SY5Y cell toxicity model. SH-SY5Y cells were seeded at 1 × 10^4^ cells per well and maintained under the culture conditions described above. After 24 h, the cells were pretreated with the test samples for 1 h, followed by exposure to 1.25 µM of Aβ_42_ for an additional 24 h. Control cells received complete medium containing 0.5% DMSO. Cell viability was subsequently assessed using the CCK-8 assay described previously. The experiment comprised three independent biological replicates, with each replicate analyzed in triplicate [46].

#### 4.10.4. Effects of *C. asiatica* Extract, Asiaticoside, and Asiatic Acid on TNF-α, IL-18, HO-1, and Caspase-3 Expression in Aβ_42_-Induced SH-SY5Y Cells

The levels of the neuroinflammatory cytokines tumor necrosis factor-α (TNF-α) and interleukin-18 (IL-18), along with heme oxygenase-1 (HO-1) and caspase-3, were quantified in SH-SY5Y cells using commercially available detection kits. Briefly, the cells were stimulated with Aβ_42_ and subsequently treated with *C. asiatica*, AS, or AA for 24 h. Following incubation, the culture supernatants were collected and centrifuged, and the concentrations of the target analytes were determined in accordance with the respective manufacturers’ protocols [47].

### 4.11. Molecular Docking

The crystal structures of TNF-α (PDB: 2AZ5 [39]), IL-18 (PDB: 3WO2 [40]), HO-1 (PDB: 3HOK [41]), and caspase-3 (PDB: 6CL0 [42]) were selected as the target proteins for the molecular docking analysis. Protein and ligand preparation was conducted according to the procedure reported in a previous study [38]. Briefly, the co-crystallized ligands were separated from their respective protein structures using Discovery Studio Visualizer, and the resulting protein and ligand files were saved in PDB format. Molecular docking was performed with AutoDock 4.0 using 50 independent docking runs and a grid size of 50 × 50 × 50 points. To validate the docking protocol, a re-docking procedure was performed using each native co-crystallized ligand. The separated ligands were re-docked into their corresponding binding sites, and the resulting poses were compared with the experimentally determined binding orientations. Grid parameters yielding the lowest root-mean-square deviation (RMSD) values—below 2 Å—were considered suitable for subsequent analyses. Each test compound was then docked using the same parameters established for its corresponding native ligand. The most favorable binding conformations were evaluated based on their predicted binding free energies (ΔG) and estimated inhibition constants (Ki).

### 4.12. Frontier Molecular Orbital (FMO) Calculations

Avogadro software (version 1.2.0) was used to construct the initial molecular structures [48]. The optimized Cartesian coordinates were subsequently obtained and used to generate input files for ORCA calculations in the .inp format [49]. Geometry optimization was performed using the restricted Hartree–Fock (RHF) method with the def2-SVP basis set. The ORCA calculations were executed from an administrator command prompt by navigating to the directory containing the input files and running the following command:

Orca filename.inp > filename.out

Molden-format files were then generated from the same working directory using:

Orca_2mkl filename-molden

The resulting files were visualized using IboView (version 20211019) [50]. This software was used to perform intrinsic bond orbital (IBO) calculations and to examine the optimized molecular geometries and molecular-orbital (MO) representations.

### 4.13. Assessment of Drug–Likeness, In Silico ADMET and Toxicity Prediction

Drug-likeness and ADMET profiles were assessed using the online SwissADME platform (http://www.swissadme.ch; accessed 20 July 2026) [32]. The Simplified Molecular Input Line Entry System (SMILES) representations of all ligands were retrieved from the PubChem database and used as input for the analyses. Furthermore, Protox-III was utilized to predict potential toxicological effects, including hepatotoxicity, cardiotoxicity, and neurotoxicity [37].

### 4.14. Statistical Analysis

All experimental data are presented as mean ± standard deviation (SD) from a minimum of three independent replicates (*n* ≥ 3). Statistical differences among treatment groups were evaluated by one-way Analysis of Variance (ANOVA). Upon obtaining a statistically significant overall F-test (*p* < 0.05), Fisher’s Least Significant Difference (LSD) post hoc test was applied for planned pairwise comparisons. This protected LSD approach was adopted for initial biological screening; however, because Fisher’s LSD does not control family-wise error rates as conservatively as post hoc adjustments like Tukey’s HSD or Bonferroni corrections across multiple inflammatory and apoptotic endpoints, comparisons demonstrating marginal significance (*p* < 0.05) are interpreted with appropriate caution. Differences were considered statistically significant at *p* < 0.05.

## 5. Conclusions

This study demonstrates that integrating Hansen solubility prediction, green extraction, chemical characterization, and biological evaluation effectively identifies neuroprotective constituents of *C. asiatica*. A 60% ethanol–water system provided optimal recovery of AS and AA with favorable sustainability and analytical practicality. Although neither compound showed strong direct antioxidant activity, both protected Aβ_42_-challenged SH-SY5Y cells, with AA exhibiting superior anti-inflammatory, antioxidant-defense, and anti-apoptotic effects. Molecular docking results were consistent with these cellular findings, although direct experimental target validation remains necessary. To advance this research, future work should prioritize key directions, including reference-standard confirmation of AS and AA, cytotoxicity evaluation in normal cell models, pathway-specific mechanism studies, and in vivo pharmacokinetic and efficacy investigations.

## Figures and Tables

**Table 1:** Physicochemical properties of asiaticoside and asiatic acid calculated using SwissADME.

Properties	Asiatic Acid	Asiaticoside
Molecular Weight (g/mole)	488.70	959.12
Log P*_o_*_/*w*_	4.45	−0.33
TPSA (Å^2^)	97.99	315.21
Water solubility (mg/mL)	2.29 × 10^−4^	6.20 × 10^−3^
Number of H-bond acceptors	5	19
Number of H-bond donors	4	12
Lipinski	Yes, 0 violation	No, 3 violations
GI absorption	Low	No
BBB	No	No

(BBB = Blood–brain barrier; GI = Gastrointestinal; TPSA = Topological polar surface area).

**Table 2:** System suitability results of the analytical method.

Analyte	% RSD of Retention Time	% RSD of Peak Area	Resolution	Number of Theoretical Plate	Tailing Factor
Acceptance criteria	≤2%	≤2%	>2	2500	≤2
Asiaticoside	0.37	0.52	2.95	238,013	±1.11
Asiatic acid	0.05	0.34	3.42	138,336	±1.39

**Table 3:** The retention time, linear range, regression equation, coefficient of determination (R^2^), limit of detection (LOD), and limit of quantification (LOQ) for the simultaneous analysis of reference standards by HPLC (*n* = 3).

Retention Time (min)	Analyte	Range (µg/mL)		Linear Equation	R^2^	LOD (µg/mL)	LOQ (µg/mL)
				Y = 1.0564X − 2.0481	0.9996	10.00	30.29
15.71	Asiaticoside	32–1290		Y = 1.0534X + 1.6260	0.9995		
				Y = 1.0563X + 4.0312	0.9998		
			mean	Y = 1.0554X + 1.2997	0.9996		
				Y = 2.6610X − 1.1363	0.9996	5.41	16.40
23.82	Asiatic acid	32–180		Y = 2.6051X + 6.4405	0.9997		
				Y = 2.6121X + 6.2021	0.9996		
			mean	Y = 2.6261X + 3.8354	0.9995		

**Table 4:** Accuracy and precision for asiaticoside and asiatic acid in *C. asiatica* (*n* = 3).

Analyte	Spiked (µg/mL)	Day	Found (µg/mL)	SD	Intra-Day %RSD	% Recovery	Inter-Day %RSD
Asiaticoside	350	1	343.51	0.25	0.25	98.15	0.32
		2	342.09	0.89	0.91	97.74	
		3	341.37	0.89	0.91	97.53	
	500	1	487.74	0.17	0.17	97.55	0.21
		2	486.32	0.20	0.21	97.26	
		3	485.77	0.26	0.27	97.15	
	650	1	634.95	0.25	0.26	97.68	0.11
		2	633.55	0.71	0.72	97.47	
		3	634.03	0.71	0.72	97.54	
Asiatic acid	60	1	60.79	0.18	0.18	101.32	0.24
		2	60.50	0.88	0.88	100.83	
		3	60.66	1.06	1.06	101.10	
	90	1	90.69	0.59	0.59	100.77	0.72
		2	91.50	0.45	0.44	101.67	
		3	90.20	0.14	0.14	100.22	
	120	1	119.68	0.08	0.08	99.73	0.09
		2	119.46	0.88	0.88	99.55	
		3	119.58	0.72	0.72	99.65	

**Table 5:** Prediction of Hansen solubility parameters of asiaticoside.

Group	Ni	Ci			(NiCi) d	(NiCi) *p*	(NiCi) h
1st-order		**δ**D	**δ**P	**δ**H			
-CH3	7	−0.9714	−1.6448	−0.7813	−6.7998	−11.5136	−5.4691
-COO-	1	0.2039	3.4637	1.1389	0.2039	3.4637	1.1389
>CHOH	13	0.1123	0.2564	−0.1928	1.4599	3.3332	−2.5064
>CH-	22	0.645	0.6491	−0.2018	14.1900	14.2802	−4.4396
>C<	5	1.2686	2.0838	0.0866	6.3430	10.4190	0.4330
-CH=C<	1	0.5372	−0.9024	−1.8872	0.5372	−0.9024	−1.8872
-CH2	11	−0.0269	−0.3045	−0.4119	−0.2959	−3.3495	−4.5309
-O-	6	0.8833	1.6853	0.447	5.2998	10.1118	2.6820
OH	8	−0.3462	1.1404	7.1908	−2.7696	9.1232	57.5264
-C=C-	1	0.3592	1.0526	−15.4659	0.3592	1.0526	−15.4659
			ΣNiCi		18.5277	36.0182	27.4812
Group	Mj		Dj		(MiDi) d	(MiDi) *p*	(MiDi) h
2nd-order		**δ**D	**δ**P	**δ**H			
ring of 6 carbon	5	−0.3874	−3.6432	0	−1.937	−18.216	0
ring of 5 carbon	3	−0.6681	−2.343	−0.3079	−2.0043	−7.029	−0.9237
cyclic OH	5	−0.0876	−3.522	0.5914	−0.438	−17.61	2.957
			ΣMiDj		−4.3793	−42.855	2.0333
ΣNiCi					18.5277	36.0182	27.4812
ΣMiDj					−4.3793	−42.855	2.0333
Constant (C)					17.3231	7.3548	7.9793
ΣNiCi + ΣMiDj + C					31.4715	0.5180	37.4938

**Table 6:** Prediction of Hansen solubility parameters of asiatic acid.

Group	Ni	Ci			(NiCi) d	(NiCi) *p*	(NiCi) h
1st-order		**δ**D	**δ**P	**δ**H			
-CH3	6	−0.9714	−1.6448	−0.7813	−5.8284	−9.8688	−4.6878
COOH	1	−0.291	0.9042	3.7391	−0.2910	0.9042	3.7391
>CHOH	3	0.1123	0.2564	−0.1928	0.3369	0.7692	−0.5784
>CH-	7	0.645	0.6491	−0.2018	4.5150	4.5437	−1.4126
>C<	5	1.2686	2.0838	0.0866	6.3430	10.4190	0.4330
-CH=C<	1	0.5372	−0.9024	−1.8872	0.5372	−0.9024	−1.8872
-CH2	9	−0.0269	−0.3045	−0.4119	−0.2421	−2.7405	−3.7071
OH	3	−0.3462	1.1404	7.1908	−1.0386	3.4212	21.5724
-C=C-	1	0.3592	1.0526	−15.4659	0.3592	1.0526	−15.4659
			ΣNiCi		4.6912	7.5982	−1.9945
Group	Mj		Dj		(MiDi) d	(MiDi) *p*	(MiDi) h
2nd-order		**δ**D	**δ**P	**δ**H			
ring of 6 carbon	5	−0.3874	−3.6432	0	−1.937	−18.216	0
			ΣMiDj		−1.937	−18.216	0
ΣNiCi					4.6912	7.5982	−1.9945
ΣMiDj					−1.937	−18.216	0
Constant (C)					17.3231	7.3548	7.9793
ΣNiCi + ΣMiDj + C					20.0773	−3.2630	5.9848

**Table 7:** Dielectric constants and calculated Hansen solubility parameter radii (Ra) of aqueous ethanol solvents, together with the extracted contents of asiaticoside and asiatic acid.

Name	Hansen Solubility Parameters			Ra		Dielectric Constant	Extraction Contents (*n* = 3) (mg/g Dried Weight)	
Solute	**δ**D	**δ**P	**δ**H	AS	AA		AS	AA
Asiaticoside	31.4715	0.5180	37.4938					
Asiatic acid	20.0773	−3.2630	5.9848					
Solvent								
100% Ethanol	15.80	8.80	19.40	37.13	19.97	24.30	4.83 ± 0.30	2.66 ± 0.21
80% Ethanol	15.74	10.24	23.98	35.60	24.11	37.70	8.59 ± 0.99	2.84 ± 0.25
70% Ethanol	15.71	10.96	26.27	35.05	26.27	43.76	9.08 ± 0.38	2.92 ± 0.17
60% Ethanol	15.68	11.68	28.56	34.67	28.47	49.81	9.94 ± 0.24	3.26 ± 0.07
40% Ethanol	15.62	13.12	33.14	34.39	32.94	60.80	8.72 ± 0.11	1.43 ± 0.09
20% Ethanol	15.56	14.56	37.72	34.78	37.50	70.82	0	1.65 ± 0.14
0% Ethanol	15.50	16.00	42.30	35.82	42.11	80.00	0	0.92 ± 0.07

**Table 8:** Total phenolic content (TPC), total flavonoid content (TFC), and antioxidant activity of the *C. asiatica* leaf extract, together with the calculated HOMO and LUMO energies (electron volts, eV) and antioxidant activities of asiaticoside, asiatic acid, and quercetin.

Compounds	TPC mg GAE/g	TFC	HOMO (eV)	LUMO (eV)	ΔE (eV)	DPPH (IC_50_, mg/mL)
		**mg QE/g**				
Extract	7.61 ± 0.24	22.64 ± 3.71				0.12 ± 0.01
Asiaticoside			–6.3784	7.2273	13.6957	>0.66
Asiatic acid			–7.4336	2.1657	9.5993	>0.66
Quercetin			–7.8091	2.0910	9.9001	0.0018 ± 0.0001

(HOMO = highest occupied molecular orbital; LUMO = lowest unoccupied molecular orbital; ΔE = HOMO–LUMO gap).

**Table 9:** Effects of *C. asiatica* extract, asiaticoside, and asiatic acid on the viability of SH-SY5Y cells.

Samples	Concentrations	% Cell Viability (Mean ± SD, *n* = 3) *
*C. asiatica* extract	1 mg/mL	1.27 ± 3.97%
	0.1 mg/mL	81.73 ± 2.90%
	0.01 mg/mL	99.17 ± 6.45%
	0.001 mg/mL	100.27 ± 3.28%
	0.0001 mg/mL	108.76 ± 2.48%
Asiaticoside	1 µM	99.21 ± 12.31%
	0.1 µM	101.26 ± 8.39%
	0.01 µM	106.13 ± 5.99%
	0.001 µM	98.94 ± 7.88%
	0.0001 µM	99.62 ± 15.92%
Asiatic acid	1 nM	74.92 ± 11.30%
	0.1 nM	89.09 ± 8.45%
	10 pM	109.51 ± 7.40%
	1 pM	104.77 ± 5.48%
	0.1 pM	102.51 ± 9.22%

* Relative assay responses.

**Table 10:** Toxicity assessment results of asiaticoside and asiatic acid analyzed using Protox–III software.

Toxicity Profiles	Asiaticoside	Asiatic Acid
Acute Oral Toxicity (LD_50_: mg/kg)	4000	2000
Toxicity Class	5	4
Hepatotoxicity	Inactive	Inactive
Respiratory toxicity	Inactive	Inactive
Neurotoxicity	Inactive	Inactive
Cardiotoxicity	Active	Active
Nephrotoxicity	Active	Active

Note: Toxicity is in the classification form 1–6. Class 1 is the most toxic and lethal and class 6 is non–toxic.

**Table 11:** Cell viability (%) and anti-inflammatory activity, assessed by TNF-α and IL-18 production, as well as antioxidant enzyme (HO-1) and apoptotic marker (caspase-3) levels in amyloid-β-induced SH-SY5Y cells treated with asiaticoside, asiatic acid, or *C. asiatica* leaf extract. Values are expressed as mean ± SD (*n* = 3).

Samples	Concentration	Cell Viability (%)	TNF-α (pg/mL)	IL-18 (pg/mL)	HO-1 (pg/mL)	Caspase-3 (ng/mL)
DMSO	-	97.81 ± 5.95 ****	1.16 ± 0.01 *	2.43 ± 0.84 **	7.29 ± 0.98 **	0.01 ± 0.01 **
Amyloid Beta	1.25 μM	26.84 ± 5.56	1.55 ± 0.05	4.40 ± 0.79	10.80 ± 0.29	0.04 ± 0.01
Asiaticoside	10 nM	75.92 ± 2.43 ****	0.39 ± 0.11 ***	0.46 ± 0.17 ***	11.16 ± 0.16	0.04 ± 0.00
Asiatic acid	10 pM	92.81 ± 1.90 ****	0.67 ± 0.07 ***	1.75 ± 0.60 ***	16.91 ± 2.19 ***	0.02 ± 0.01 *
*C. asiatica* extract	100 ng/mL	80.21 ± 10.62 ****	0.76 ± 0.02 ***	0.56 ± 0.17***	11.68 ± 0.38	0.05 ± 0.02

Note: Statistical significance compared with the amyloid-beta-treated group: *p* < 0.05 (*), *p* ≤ 0.01 (**), *p* ≤ 0.001 (***), and *p* ≤ 0.0001 (****).

**Table 12:** Summary of the putative interactions of asiaticoside and asiatic acid with amino acid residues in the active sites of TNF-α, IL-18, HO-1, and caspase-3.

Protein Targets	Ligands	Energy of Binding (ΔG) (kcal/mol)	Ki	Amino Acid Residues Involved in Interactions
TNF-α (PDB: 2AZ5) RMSD = 1.858 Å	AS	−8.08	1.19 µM	LeuA57, TyrA59, GlnA61, TyrA119, LeuA120, GlyA121, GlyA149, TyrA151, IleA155, LeuB57, IluB58, TyrB59, TyrB119, LeuB120, GlyB121, GlyB122, ValB123, LeuD55, LeuD157
	AA	−8.60	0.50 µM	LeuA57, IleuA58, TyrA119, LeuA120, GlyA121, GlyA122, LeuB57, TyrB59, SerB60, GlyB61, TyrB119, LeuB120, GlyB121, TyrB151, LeuD55, LeuD157
IL-18 (PDB: 3WO2) RMSD = 1.591 Å	AS	−1.16	141.10 mM	LYS58, LEU9, SER10, VAL11, ARG13, ASP35, CYS38, ILE46, GLN154, ASN155, GLU156, ASP157
	AA	−4.02	1.13 mM	SER57, LYS58, LEU9, ILE48, ASN155, GLU156, ASP157
HO-1 (PDB: 3HOK) RMSD = 1.667 Å	AS	−4.41	588.10 µM	GLU32, PHE33, ARG35, ASN36, CYS38, GLN41, VAL42, VAL50, ARG136, GLY139, SER142, VAL146, LEU147, ILE150, PHE214, LEU217, GLN218, LEU221
	AA	−9.20	0.18 µM	ALA28, GLU32, VAL42, PHE47, VAL50, TYR135, ARG136, GLY139, ASP140, GLY143, LEU147, ALA151, PHE167, PHE214
Caspase-3 (PDB: 6CL0) RMSD = 1.514 Å	AS	−6.83	9.93 µM	ARGA64, SERA65, SERA120, HISA121, GLYA122, ALAA162, CYSA163, LEUA168, TYRB204, SERB205, TRPB206, ARGB207, ASNB208, SERB209, LYSB210, PHEB250, SERB251, PHEB252, ASP253
	AA	−7.95	1.49 µM	CYSA163, LEUA168, TYRB204, SERB205, TRPB206, ARGB207, ASNB208, SERB209, LYSB210, SERB249, PHEB250, SERB251, PHEB256

Note: Green indicates amino acids involved in hydrogen bonding. RMSD is the root-mean-square deviation from redocking the co-crystallized ligand in the PDB structure.

## Data Availability

The original contributions presented in this study are included in the article. Further inquiries can be directed to the corresponding author.

## Abbreviations

The following abbreviations are used in this manuscript:

AA	Asiatic acid
ADMET	Absorption Distribution Metabolism Excretion Toxicity
Ala	Alanine
AlCl_3_	Aluminum chloride
AOAC	Association of Official Analytical Collaboration
Arg	Arginine
AS	Asiaticoside
Asn	Asparagine
Asp	Aspartic acid
BAGI	Blue Applicability Grade Index
C18	Octadecylsilane
COX-2	Cyclooxygenase-2
Cys	Cysteine
DPPH	2,2-Diphenyl-1-picrylhydrazyl
HPLC	High-Performance Liquid Chromatography
°C	Celsius degree
cm	Centimeter
EtOH	Ethanol
eV	Electron volt
FMO	Frontier molecular orbital
GAE	Gallic acid equivalents
GI	Gastrointestinal
Gln	Glutamine
Glu	Glutamic acid
Gly	Glycine
His	Histidine
HOMO	Highest occupied molecular orbital
HO-1	Heme oxygenase-1
HSP	Hansen solubility parameter
IC_50_	Half-maximal inhibitory concentration
ICH	The International Council for Harmonisation of Technical Requirements for Pharmaceuticals for Human Use
IL-1β	Interleukin-1beta
IL-18	Interleukin-18
Ile	Isoleucine
Kcal	Kilocalorie
Ki	Inhibition constant
LOD	Limit of Detection
LOQ	Limit of Quantitation
LOX	Lipoxygenase
Leu	Leucine
LUMO	Lowest unoccupied molecular orbital
mM	Millimolar
mL	Milliliter
Mol	Mole
Na_2_CO_3_	Sodium carbonate
nM	Nanomolar
PDA	Photo Diode-Array
PDB	Protein data bank
PGE2	Prostaglandin E2
Phe	Phenylalanine
pM	Picomolar
QE	Quercetin equivalent
R^2^	Coefficient of determination
Ra	Hansen solubility parameter distance
RMSD	Root means square deviation
RT	Retention time
SD	Standard deviation
Ser	Serine
TNF-α	Tumor necrosis factor-alpha
TPSA	Topological polar surface area
Trp	Tryptophan
Tyr	Tyrosine
UAE	Ultrasound-assisted extraction
Val	Valine
ΔG	Free Energy of Binding
δD	Dispersion force
δH	Hydrogen bonding energy
δP	Dipolar intermolecular force
µg	Microgram
%RSD	Percentage relative standard deviation
λmax	Wavelength of Maximum Absorbance
